# AMPK protects proximal tubular epithelial cells from lysosomal dysfunction and dedifferentiation induced by lipotoxicity

**DOI:** 10.1080/15548627.2024.2435238

**Published:** 2024-12-15

**Authors:** Louise Pierre, Florian Juszczak, Valentine Delmotte, Morgane Decarnoncle, Benjamin Ledoux, Laurent Bultot, Luc Bertrand, Marielle Boonen, Patricia Renard, Thierry Arnould, Anne-Emilie Declèves

**Affiliations:** a Laboratory of Biochemistry and Cell Biology, Namur Research Institute for Life Sciences (NARILIS), University of Namur, Namur, Belgium; b Laboratory of Metabolic and Molecular Biochemistry, Faculty of Medicine and Pharmacy, Research Institute for Health Sciences and Technology, University of Mons, Mons, Belgium; c Pole of Cardiovascular Research, Experimental and Clinical Research Institute (CARD), UCLouvain, Brussels, Belgium; d WELBIO Department, WEL Research Institute, Wavre, Belgium; e URPhyM, Intracellular Trafficking Biology, NARILIS, University of Namur, Namur, Belgium

**Keywords:** Autophagy, chronic kidney disease, lipid accumulation, obesity, proximal tubules, AMPK

## Abstract

Renal proximal tubules are a primary site of injury in metabolic diseases. In obese patients and animal models, proximal tubular epithelial cells (PTECs) display dysregulated lipid metabolism, organelle dysfunctions, and oxidative stress that contribute to interstitial inflammation, fibrosis and ultimately end-stage renal failure. Our research group previously pointed out AMP-activated protein kinase (AMPK) decline as a driver of obesity-induced renal disease. Because PTECs display high macroautophagic/autophagic activity and rely heavily on their endo-lysosomal system, we investigated the effect of lipid stress on autophagic flux and lysosomes in these cells. Using a model of highly differentiated primary PTECs challenged with palmitate, our data placed lysosomes at the cornerstone of the lipotoxic phenotype. As soon as 6 h after palmitate exposure, cells displayed impaired lysosomal acidification subsequently leading to autophagosome accumulation and activation of lysosomal biogenesis. We also showed the inability of lysosomal quality control to restore acidic pH which finally drove PTECs dedifferentiation. When palmitate-induced AMPK activity decline was prevented by AMPK activators, lysosomal acidification and the differentiation profile of PTECs were preserved. Our work provided key insights on the importance of lysosomes in PTECs homeostasis and lipotoxicity and demonstrated the potential of AMPK in protecting the organelle from lipid stress.

**Abbreviation**: ACAC: acetyl-CoA carboxylase; ACTB: actin beta; AICAR: 5-aminoimidazole-4-carboxamide-1-β-D-ribofuranoside; AMPK: AMP-activated protein kinase; APQ1: aquaporin 1 (Colton blood group); BSA: bovine serum albumin; CDH16: cadherin 16; CKD: chronic kidney disease; CTSB: cathepsin B; CTSD: cathepsin D; EPB41L5: erythrocyte membrane protein band 4.1 like 5; EIF4EBP1: eukaryotic translation initiation factor 4E binding protein 1; EMT: epithelial-to-mesenchymal transition; FA: fatty acid; FCCP: carbonyl cyanide 4-(trifluoromethoxy)phenylhydrazone; GFP: green fluorescent protein; GUSB: glucuronidase beta; HEXB: hexosaminidase subunit beta; LAMP: lysosomal associated membrane protein; LD: lipid droplet; LGALS3: galectin 3; LLOMe: L-leucyl-L-leucine methyl ester hydrobromide; LMP: lysosomal membrane permeabilization; LRP2: LDL receptor related protein 2; LSD: lysosomal storage disorder; MAP1LC3/LC3: microtubule associated protein 1 light chain 3; MCOLN1: mucolipin TRP cation channel 1; MG132: N-benzyloxycarbonyl-L-leucyl-L-leucyl-L-leucinal; MmPTECs: Mus musculus (mouse) proximal tubular epithelial cells; MTORC1: mechanistic target of rapamycin kinase complex 1; OA: oleate; PA: palmitate; PIKFYVE: phosphoinositide kinase, FYVE-type zinc finger containing; PTs: proximal tubules; PTECs: proximal tubular epithelial cells; PRKAA: protein kinase AMP-activated catalytic subunit alpha; RFP: red fluorescent protein; RPS6KB: ribosomal protein S6 kinase B; SLC5A2: solute carrier family 5 member 2; SOX9: SRY-box transcription factor 9; SQSTM1: sequestosome 1; TFEB: transcription factor EB; Ub: ubiquitin; ULK1: unc-51 like autophagy activating kinase 1; VIM: vimentin

## Introduction

Proximal tubular epithelial cells (PTECs) are a primary site of metabolic injury because of high metabolic rates [1]. They require a large amount of ATP for active electrolyte-transporting processes, mostly provided by the mitochondrial free fatty acid (FA) oxidation [1]. Those are taken up in PTECs by endocytosis of albumin-bound FAs [2] or by transporters such as SLC7A2/FATP2 (solute carrier family 27 member 2) [3] and the translocase CD36 [4]. In diabetic chronic kidney disease (CKD), a tubulocentric view has emerged postulating that tubulopathy is the primary injury and a causative event in the development of the disease [5]. This concept emerged since some diabetic patients with renal failure present normoalbuminuria, and then was further supported by the occurrence of tubule injuries (stress marker expression and tubular proteinuria) prior glomerular damage in animal models of diabetes [6,7]. Tubular biomarkers have thus gained paramount importance for early diagnosis of the disease. Diabetes-related kidney disease shared several pathological features with obesity-induced CKD. Indeed, obesity is now recognized as the second most predictive factor for end-stage renal disease, independently of diabetes and hypertension [8]. Several studies showed the direct contribution of obesity on renal lipid metabolism dysregulation, defined as lipotoxicity [9–11]. In line with the tubulocentric view, PTECs of both patients and animal models with obesity-related CKD present ectopic lipid depositions associated with tubulointerstitial inflammation and fibrosis [12–15]. Lipid droplet (LD) accumulation is concomitant with mitochondrial damage, autophagic and lysosomal marker changes in PTECs, which ultimately lead to brush border alterations and loss of polarity [13].

AMP-activated protein kinase (AMPK) was highlighted as a key mediator in the development and progression of obesity-induced CKD. The enzyme is abundantly expressed in kidneys, but its renal activity is decreased in response to metabolic stresses. Indeed, impaired AMPK activity is described in animal models of metabolic CKD induced by high fat diet [15] or diabetes [16] as well as in patients with diabetic nephropathy [17]. In contrast, we showed that its activation, by 5-aminoimidazole-4-carboxamide-1-β-D-ribofuranoside (AICAR) supplementation [11,13] or exercise training [14], protects renal function and structure in obese animals and prevents the development of CKD. In addition, the protection of obese female mice (over males) against the development of CKD was recently shown to be mediated by AMPK activity [18]. AMPK is a ubiquitous serine/threonine kinase consisting of a catalytic PRKAA/α subunit and two regulatory PRKAB/β and PRKAG/γ subunits [19]. It is the master energy sensor of eukaryotic cells and is crucial in PTECs due to their elevated metabolic requirements. Previous studies from our group highlighted a strong correlation between AMPK activation and reduced PTEC vacuolization [11,13,14,18]. In addition, lean mice deficient for the PRKAB1/β1 subunit display LD accumulation in PTECs specifically [20], reinforcing that impaired AMPK activity results in dysregulated lipid metabolism in PTECs.

Macroautophagy/autophagy is the process by which cytoplasmic cargos are engulfed in a double-membrane structure, named a phagophore, that matures into an autophagosome, which delivers the cargo to lysosomes for degradation and recycling [21]. PTECs display high autophagic activity and thus rely heavily on lysosomal function [21]. Moreover, the degradation of filtered proteins after reabsorption by receptor-mediated endocytosis also occur in the lysosomes [22]. In PTs of obese mice, we [13,14] and others [12] showed the accumulation of enlarged lysosomes and dysregulation of autophagic markers, both prevented by AMPK activation. These changes may result from increased formation and/or decreased clearance of autophagic vacuoles and were thus hard to interpret. Activation of autophagy in response to lipotoxicity has been demonstrated in PTECs with a role in the degradation of cell components damaged by oxidative stress [12]. This elevated initiation is thought to finally place an important burden on lysosomal degradation leading to saturation. It is also known that autophagosome accumulation could result from defective fusion with lysosomes [23]. Recently, it was shown that cultured hepatocytes and cardiomyocytes exposed to FAs display direct lysosomal damage, in an autophagy-independent manner [24–26]. Interestingly, several evidence suggest a strong interaction between lysosomes and AMPK as its non-canonical activation is regulated by the lysosomal V-ATPase-Ragulator complex [27]. Moreover, some of its downstream targets are involved in lysosomal biogenesis [28] and maintenance of lysosomal ion homeostasis [29]. Overall, the effects of lipid overload on lysosomes in PTECs need to be clarified, especially regarding the mechanisms of AMPK protection. Once damaged, lysosomes might be either repaired, removed or replaced by several pathways to ensure cell homeostasis [30]. The activation of these pathways remains to be determined in response to lysosomal stress induced by lipotoxicity. Moreover, given the importance of lysosomal homeostasis for PTECs, the effect of lipotoxicity on their function of protein endocytosis is of great interest.

In this work, using FA-exposed PTECs, we demonstrate that the inhibition of autophagic flux is a consequence of lysosomal dysfunction induced by FA overload. It leads to the activation of lysosomal quality control pathways and finally drives cell dedifferentiation. Interestingly, AMPK activation maintains lysosomal homeostasis and is partly protective against FA-induced PTECs dysfunction.

## Results

### Treatment with palmitate leads to autophagosome accumulation in mouse proximal tubular epithelial cells

**Figure 1.**
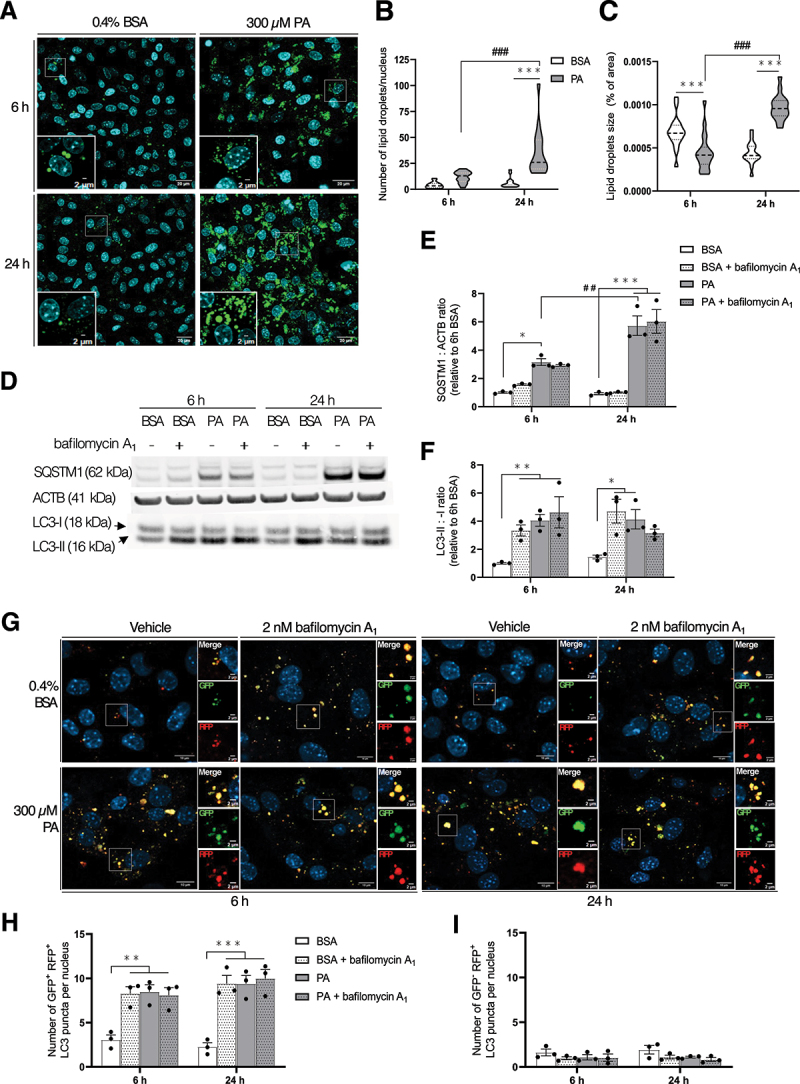
PA induces the accumulation of autophagosomes in *Mus musculus* (mouse) proximal tubular epithelial cells (MmPTECs). (A) MmPTECs were treated with 300 µM PA or 0.4% BSA for 6 or 24 h and stained with BODIPY^TM^ 493/503 for 15 min. (B, C) Quantifications of lipid droplet (B) number and (C) size on 100 cells per condition by lipid droplets MRI tool. (D) Representative western blot and (E,F) quantitative densitometry analysis of LC3, SQSTM1 and ACTB/β-actin in MmPTECs treated with 300 µM PA or 0.4% BSA for 6 or 24 with or without 2 nM bafilomycin A_1_ for last 6 h. (G) Representative micrographs of MmPTECs expressing mRFP-GFP-LC3B and treated with 300 µM PA or 0.4% BSA for 6 or 24 h with or without 2 nM bafilomycin A_1_ for the last 6 h. GFP^+^ RFP^+^ (yellow) puncta indicate autophagosomes (neutral pH), and GFP^−^ RFP^+^ (red) ones indicate acidic pH. Quantifications of the number of (H) GFP^+^ RFP^+^ and (I) GFP^−^ RFP^+^ puncta on 30 cells per group. Data are represented as (B, C) means and quarters or as (E, F, H, I) means ± SEM of three independent biological experiments. Statistical analyses were performed by two-way ANOVA followed by (F, H, I) Dunnett’s or (B, C, E) Tukey’s post-hoc test.

Changes in autophagic activity secondary to lipid stress have been shown in various PTECs models even if conflicting results and interpretations remain [12–14]. To further investigate how this pathway is affected upon lipid challenge, primary *Mus musculus* (mouse) PTECs (MmPTECs) were exposed to palmitate (PA), widely used to mimic lipotoxicity *in vitro*. Unlike other models of cultured PTECs, these cells display high differentiation state [22]. The concentration of 300 µM PA was selected in this work as it induced LD accumulation but did not significantly impair cell viability (Figure S1). PA-treated cells displayed elevated LD number (Figure 1A,B) and LD size (Figure 1A,C) after 24 h when compared with cells treated with 0.4% bovine serum albumin (BSA) used as vehicle control.

*p ≤ 0.05; **p ≤ 0.01; ***p ≤ 0.001 *versus* corresponding BSA group; ##p ≤ 0.01; ###p ≤ 0.001 between PA groups.

After 6 and 24 h of PA treatment, the MAP1LC3/LC3 (microtubule associated protein 1 light chain 3)-II:LC3-I ratio and SQSTM1/p62 (sequestosome 1) abundance were significantly elevated (Figure 1D-F). *Sqstm1* relative mRNA abundance was also significantly increased in PA-treated cells (Figure S2A) which make the interpretation of SQSTM1 changes difficult in the context of autophagy. The LC3-II:-I ratio was not further increased upon the addition of bafilomycin A_1_, a lysomotropic compound inhibiting lysosomal acidification [31] (Figure 1D,F). This suggests an impairment of autophagosome turnover in PTECs exposed to PA. To confirm this hypothesis, we monitored the autophagic flux with a construct encoding a cDNA of *LC3B* fused with pH-sensitive green fluorescent protein (GFP) and pH-insensitive red fluorescent protein (RFP) [32]. In these cells, 6 and 24 h of PA treatment significantly increased the number of autophagosomes at neutral pH (GFP^+^ RFP^+^ puncta) (Figure 1G,H). Still, the addition of bafilomycin A_1_ had no effect on PA-treated cells. In contrast, the number of acidic autolysosomes (GFP^−^ RFP^+^ puncta) was unchanged between PA- and BSA-treated cells (Figure 1G,I). Altogether, these data highlight that autophagosome degradation is quickly impaired in MmPTECs upon lipid challenge which is in accordance with the literature [12]. However, no change in the initiation was observed upon PA treatment. The phosphorylation of ULK1 (unc-51 like autophagy activating kinase 1) at Ser555, a marker of autophagy activation [33], was not significantly affected by PA from 3 to 24 h in MmPTECs (Figure S2B,C). Absence of PA-induced initiation was reinforced by the similar effect of BSA and bafilomycin A_1_, PA with and without bafilomycin A_1_ on the LC3-II:-I ratio (Figure 1D,F) and the number of GFP^+^ RFP^+^ LC3 puncta (Figure 1G,H).

### AMPK pharmacological activation prevents PA-induced autophagic flux inhibition in MmPTECs

**Figure 2.**
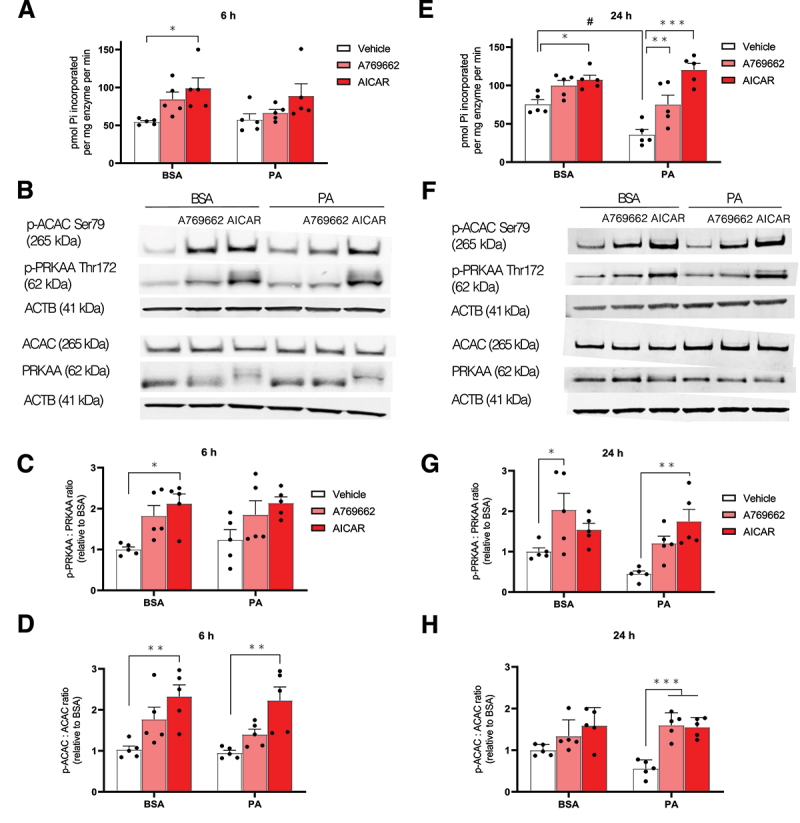
PA alters AMPK activity and signalization after 24 h in MmPTECs. (A, E) AMPK was immunoprecipitated by anti-PRKAA1/AMPKα1 and anti-PRKAA2/AMPKα2 antibodies in lysates from MmPTECs previously treated for (A) 6 or (E) 24 h with 300 µM PA or 0.4% BSA with or without 100 µM A769662 or 2 mM AICAR. Phosphotransferase activity was assessed toward the AMARA peptide. Data are presented as the means of pi (pmol) incorporated per µg of proteins per min ± SEM. (B, F) Representative western blot of p-ACAC (Ser79), p-PRKAA (Thr172), ACAC, AMPK and ACTB in MmPTECs treated with 300 µM PA or 0.4% BSA with or without 100 µM A769662 or 2 mM AICAR for (B) 6 or (F) 24 h. (C, D, G, H) Quantitative densitometry analysis of the p-PRKAA:PRKAA (C for 6 h, G for 24 h) and the p-ACAC:ACAC (D for 6 h, H for 24 h) ratios. Data are presented as means ± SEM of five independent biological experiments. Statistical analyses were performed by two-way ANOVA followed by Tukey’s post-hoc test.

Impaired AMPK activity is reported in different models of metabolic CKD as well as in patients presenting diabetic nephropathy [15–17]. In high fat diet-fed mice, we showed the contribution of this event to the development of CKD [13]. The present work aimed to assess the direct effect of FA overload on AMPK activity in PTECs. After a 6 h PA-treatment, AMPK activity was not affected as evidenced by unchanged activity (Figure 2A) and phosphorylation state of the enzyme on Thr172 (Figure 2B,C). AMPK-dependent phosphorylation of ACAC (acetyl-CoA carboxylase) on Ser79 was also comparable between PA- and BSA-treated mPTECs after 6 h (Figure 2B,D). However, the AMPK phosphotransferase activity became significantly impaired after 24 h of a PA treatment (Figure 2E) while AMPK and ACAC phosphorylations were slightly decreased at that time in PA-treated cells (Figure 2F-H). These results suggested that MmPTECs exhibit reduced AMPK activity and signaling when exposed to PA for an extended period. AMPK has an inhibitory action on MTORC1 activity through phosphorylation of RAPTOR and tuberous sclerosis complex 2 [34]. We thus analyzed MTORC1 activity by assessing the phosphorylation of substrates such as EIF4EBP1 (eukaryotic translation initiation factor 4E binding protein 1) on Thr37/46 and RPS6KB (ribosomal protein S6 kinase B) on Thr389. The phosphorylation states of these two proteins were significantly increased in PA-treated cells for 24 h (Figure S3). These data indicate that 24 h of PA-treatment impairs AMPK activity which is concomitant with elevated MTORC1 activity in MmPTECs.

*p ≤ 0.05; **p ≤ 0.01; ***p ≤ 0.001 in PA or BSA treatment groups; #p ≤ 0.05 between groups.

**Figure 3.**
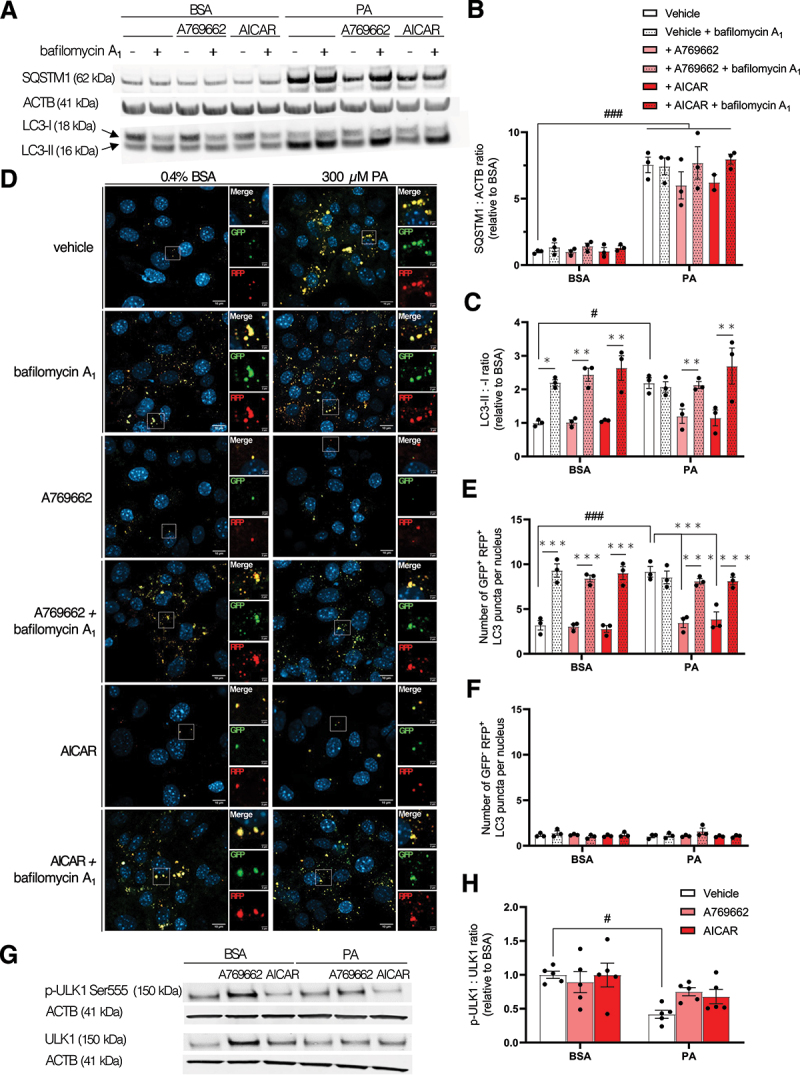
AMPK activation prevents PA-induced autophagosome accumulation in MmPTECs. (A) Representative western blot and (B, C) quantitative densitometry analysis of LC3, SQSTM1 and ACTB in MmPTECs treated for 24 h with 300 µM PA or 0.4% BSA, in the presence of 100 µM A769662 or 2 mM AICAR and with or without 2 nM bafilomycin A_1_ for last 6 h. (D) Representative micrographs of MmPTECs expressing mRFP-GFP-LC3B and treated for 24 h in the same conditions. GFP^+^ RFP^+^ (yellow) puncta indicate autophagosomes (neutral pH), and GFP^−^ RFP^+^ (red) ones indicate acidic pH. (E, F) Quantifications of the number of (E) GFP^+^ RFP^+^ and (F) GFP^−^ RFP^+^ puncta on 30 cells per group. (G) Representative western blot and (H) quantitative densitometry analysis of p-ULK1 (Ser555), ULK1 and ACTB in MmPTECs treated for 24 h with 300 µM PA or 0.4% BSA in the presence of 100 µM A769662 or 2 mM AICAR. Data are presented as means ± SEM of three or five independent biological experiments as indicated on the charts. Statistical analyses were performed by two-way ANOVA and Tukey’s post-hoc test.

Once activated, AMPK promotes autophagy by acting at multiple steps of the flux, from initiation to fusion between autophagosomes and lysosomes [35]. Therefore, its inhibition in PA-treated MmPTECs could contribute to the autophagosome accumulation observed in these cells. To test this hypothesis, we used two pharmacological AMPK activators, A769662 and AICAR [36]. We first validated their effect on AMPK activity and the phosphorylation of AMPK and its target ACAC. After 6 h, the ACAC phosphorylation was significantly elevated in the presence of AICAR in PA-treated cells (Figure 2D). After 24 h, the two activators significantly increased AMPK activity and ACAC phosphorylation in PA-treated cells while AMPK phosphorylation was significantly increased with AICAR (Figure 2E-H). These data highlighted that AMPK is activated in response to either A769662 or AICAR in PA-treated MmPTECs, an effect that is stronger after 24 h. Next, we analyzed whether the increase in LC3-II:-I ratio induced by PA treatment of 24 h could be corrected by AMPK activation, and found that it was (Figure 3A,C). However, AMPK activation did not modify the abundance of SQSTM1 in these experimental conditions (Figure 3A,B). It is likely explained by the PA-induced increased mRNA relative expression as previously shown (Figure S2A). In accordance with the LC3-II:-I ratio, the number of autophagosomes with neutral pH (GFP^+^ RFP^+^ puncta) was also reduced in PA-treated cells in the presence of AMPK activators (Figure 3D,E). Again, the number of autophagosomes with acidic pH (GFP^−^ RFP^+^ puncta) was unchanged in these conditions (Figure 3D,F). Notably, in MmPTECs exposed to both PA and the activators, bafilomycin A_1_ further increased the LC3 ratio (Figure 3A,C) as well as the number of autophagosomes with neutral pH (GFP^+^ RFP^+^ puncta) (Figure 3D,E). Comparable results were obtained when MmPTECs were incubated with PA, AMPK activators and bafilomycin A_1_ for 6 h (Figure S4). These results reveal that maintenance of AMPK activity in MmPTECs exposed to PA relieves the autophagic flux inhibition and prevents the accumulation of autophagosomes. In contrast, AMPK activators did not modulate the phosphorylation of ULK1 in PA-treated cells (Figure 3G,H), suggesting that the protection is not mediated by an activation of the initiation of autophagy.

*p ≤ 0.05; **p ≤ 0.01; ***p ≤ 0.001 in PA or BSA treatment groups; #p ≤ 0.05; ###p ≤ 0.001 between groups.

### Accumulating autophagosomes in response to PA contain ubiquitin-positive aggregates

Targeted autophagy is the process by which autophagosomes engulf selectively damaged organelles and/or cell components after cargo recognition [37]. Several types of selective autophagy were studied in the present work in MmPTECs after lipid challenge. Because PA was demonstrated to impair mitochondrial membrane potential in PTEC cell line [12], mitophagy was assessed by colocalizing LC3 with mitochondrial oxidative phosphorylation (OXPHOS) markers. However, no change in colocalization percentages was detected between PA- and BSA-treated cells for 24 h (Figure S5A,C). Next, another type of selective autophagy targeting LDs, namely lipophagy, was assessed. The impact of lipotoxicity on lipophagy is still controversial in the literature: some studies on cultured hepatocytes postulated that it is activated in response to PA [38,39] while contradictory findings on cultured β-cells were observed [40]. Lipophagy was assessed by colocalizing LC3 with PLIN2 (perilipin 2) and colocalization percentages were comparable between PA- and BSA-treated cells (Figure S5B,D).

**Figure 4.**
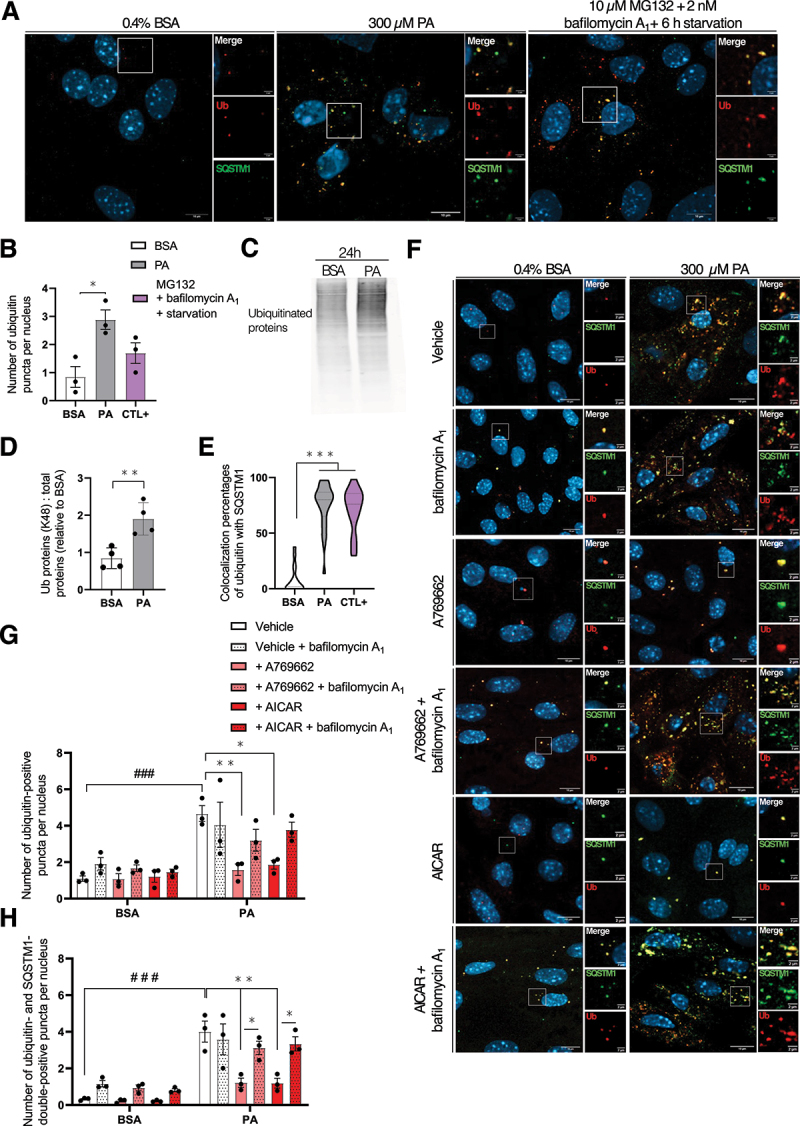
Autophagosomes in response to PA contain ubiquitin-positive aggregates in MmPTECs. (A) Representative micrographs of mPTECs treated for 24 h with 300 µM PA, 0.4% BSA or for 6 h with 10 µM MG132 and 2 nM bafilomycin A_1_ (positive control), fixed and immuno-stained for ubiquitin (red) and SQSTM1 (green). (B) Quantifications of the number of ubiquitin puncta per nucleus on 30 cells per group. (C) Representative western blot and (D) quantitative densitometry analysis of ubiquitinylated proteins normalized by total proteins in MmPTECs treated with 300 µM PA or 0.4% BSA for 24 h. (D) Quantifications of the percentages of colocalization of ub with SQSTM1 calculated by Mander’s correlation coefficients on 30 cells per group. (F) Representative micrographs of MmPTECs treated for 24 h with 300 µM PA or 0.4% BSA in the presence of 100 µM A769662 or 2 mM AICAR with and without and 2 nM bafilomycin A_1_ for last 6 h, fixed and immuno-stained for ubiquitin (red) and SQSTM1 (green). (G, H) Quantifications of the number of (G) ubiquitin puncta and (H) ubiquitin- and SQSTM1-double-positive puncta per nucleus calculated with the objects-based method on 30 cells per group. Data are presented as (B, D, G, H) means ± SEM or as (E) means and quarters of three or four independent biological experiments as indicated on the charts. Statistical analyses were performed by (D) Student’s unpaired t-test, (B, E) one-way ANOVA followed by Dunnett’s post-hoc test or (G, H) two-way ANOVA followed by Tukey’s post-hoc test.

Perez and collaborators nicely demonstrated that PA alters the endoplasmic reticulum by increasing the saturation of membrane FAs in fibroblast-induced renal epithelial cells [41]. This stress might lead to protein misfolding, which will translocate in the cytosol by the endoplasmic reticulum-associated protein degradation system. In addition, folded proteins might be directly damaged by toxic lipid intermediates and/or elevated oxidative stress, two features of lipotoxicity [42]. Cytosolic accumulation of aberrant proteins might form aggregates which, upon ubiquitination, are recognized by SQSTM1 to be engulfed in autophagosomes, a process named aggrephagy [43]. After 24 h, PA-treated cells showed significant higher number of ubiquitin (Ub) aggregates (>0.5 µm^2^) compared to BSA-treated cells (Figure 4A,B). The effect of PA was even higher than in the positive control where cells were starved and treated for 6 h with 10 µM of the proteasomal inhibitor N-benzyloxycarbonyl-L-leucyl-L-leucyl-L-leucinal (MG132) and bafilomycin A_1_ (Figure 4A,B). The significant increase in ubiquitinated protein abundance in response to 24 h of PA treatment was confirmed by western blot analysis (Figure 4C,D). In addition, colocalization percentages between Ub and SQSTM1 were significantly higher in PA-treated cells compared to BSA-treated cells after 24 h (Figure 4A,E). Comparable results were obtained after a period of PA incubation of 6 h (Figure S6). The detection of numerous Ub aggregates translates impairment of protein homeostasis in MmPTECs treated with PA. It is in accordance with previous report showing increased Ub staining in proximal tubules of obese mice [12]. The present work addresses the fate of these aggregates, that are delivered to the autophagy machinery for lysosomal degradation.

*p ≤ 0.05; **p ≤ 0.01; ***p ≤ 0.001; ###p ≤ 0.001 between groups.

Given that AMPK activation protected against autophagosomes accumulation in PA-treated cells (Figure 3), we asked about its potential protection toward Ub-tagged aggregates. AMPK pharmacological activation in PA-treated cells was associated with significantly reduced number of Ub puncta (Figure 4F,G) and puncta positive for Ub and SQSTM1 staining after 24 h (Figure 4F,H). Because the reduction of both markers was abolished with bafilomycin A_1_ in these conditions, we can conclude that AMPK activation restore the autophagic degradation of these aggregates in PA-treated cells.

### Lysosomal function is impaired by PA and protected by AMPK pharmacological activation in MmPTECs

**Figure 5.**
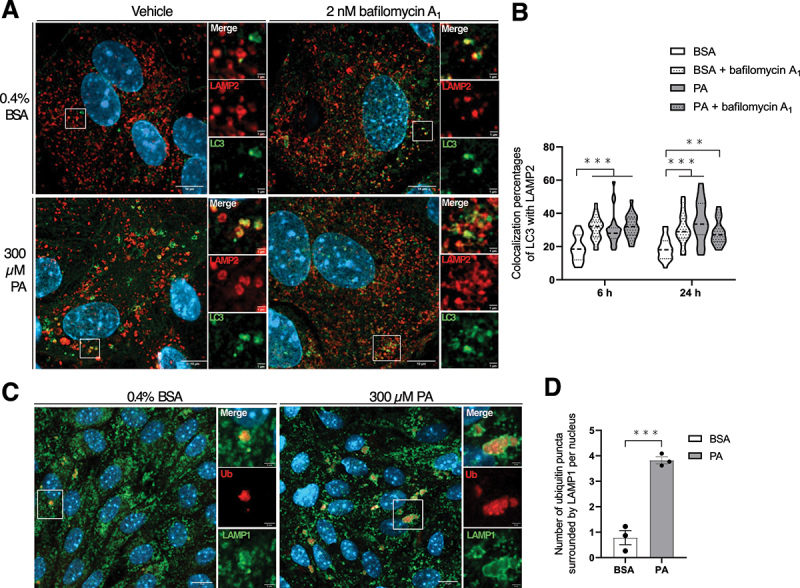
PA does not alter the fusion between autophagosomes and lysosomes in MmPTECs. (A) Representative micrographs of MmPTECs treated for 6 or 24 h (only 24 h is represented) with 300 µM PA or 0.4% BSA with and without 2 nM bafilomycin A_1_ for last 6 h, fixed and immuno-stained for LAMP2 (red) and LC3 (green). (B) Quantification of the colocalization percentages of LC3 with LAMP2 calculated by Mander’s correlation coefficients on 30 cells per group. (C) Representative micrographs of MmPTECs treated for 24 h with 300 µM PA or 0.4% BSA, fixed and immuno-stained for ubiquitin (red) and LAMP1 (green). (D) Quantification of the number of ub puncta surrounded by LAMP1 staining on 30 cells per group. Data are presented as (B) means and quarters or as (D) means ± SEM of three independent biological experiments. Statistical analyses were performed by (D) Student’s unpaired t-test or by (B) two-way ANOVA followed by Dunnett’s post-hoc test.

We have shown so far that lipotoxicity inhibits the autophagic flux while the initiation might be unaffected in MmPTECs. In this regard, it is possible that the accumulation of autophagosomes in response to PA results from defective fusion with lysosomes as demonstrated for other cell types [23,44,45]. To test this hypothesis, fusion events were evaluated by the colocalization percentages between LC3 and lysosomes through LAMP2 (lysosomal associated membrane protein 2) staining. After 6 and 24 h, the colocalization percentages were significantly higher in PA-treated cells compared with BSA-treated cells (Figure 5A,B). These results suggested that the autophagosomes observed in PA-treated cells are positive for the LAMP2 marker and are thus most likely autolysosomes. It is reinforced by the fact that Ub aggregates were engulfed in LAMP1-positive structures indicative of lysosomes (Figure 5C,D). Notably, lysosomes appeared enlarged in PA-treated cells after 24 h (Figure 5C). Because the number and the size of autophagosomes (Figure 1G,H) and their colocalization with lysosomes (Figure 5A,B) were similar between PA-treated cells and those treated with BSA and bafilomycin A_1_, we postulated that PA might act similarly to bafilomycin A_1_ and inhibit lysosomal function. In this regard, autophagosome accumulation might be a consequence of defective lysosomal degradation secondary to lipotoxicity in MmPTECs.

**p ≤ 0.01; ***p ≤ 0.001 between groups.

**Figure 6.**
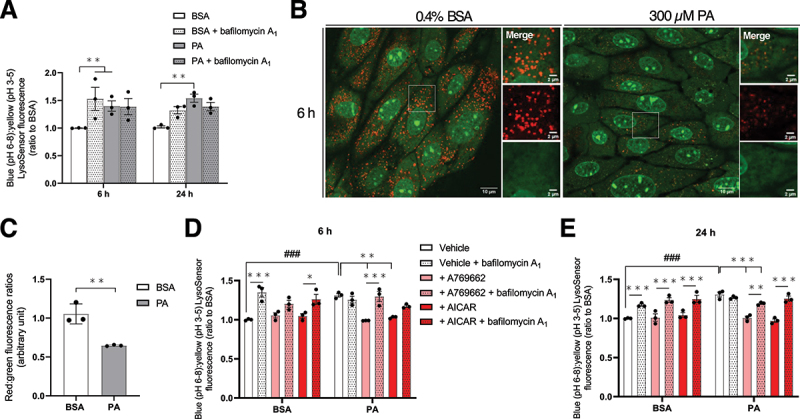
PA impairs lysosomal acidification in MmPTECs which is prevented by AMPK activation. (A) Blue (neutral pH):yellow (acidic pH) fluorescence ratios from MmPTECs treated for 6 or 24 h with 300 µM PA or 0.4% BSA with or without 2 nM bafilomycin A_1_ for last 6 h and incubated with 5 µM LysoSensor^TM^ yellow/blue DND-160 for 3 min. (B) Representative micrographs of MmPTECs treated for 6 h with 300 µM PA or 0.4% BSA and incubated with 2.5 µM acridine orange for 15 min. (C) Quantification of the red:green fluorescence ratios on 30 cells per group. (D, E) Blue (neutral pH):yellow (acidic pH) fluorescence ratios from MmPTECs treated for (D) 6 or (E) 24 h with 300 µM PA or 0.4% BSA in the presence of 100 µM A769662 or 2 mM AICAR with or without 2 nM bafilomycin A_1_ for last 6 h and stained with LysoSensor^TM^ yellow/blue DND-160. Data are presented as means ± SEM of three independent biological experiments. Statistical analyses were performed by (C) Student’s unpaired t-test or by (A, D, E) two-way ANOVA followed by (A) Dunnett’s or (D, E) Tukey’s post-hoc test.

As acidic pH is crucial for lysosomal enzyme activity, this parameter was next monitored as a marker of organelle function. The LysoSensor^TM^ Yellow/Blue DND-160 dye was used in this purpose. It stains lysosomes and emits yellow fluorescence in acidic condition (pH: 3–5) and blue fluorescence at neutral pH (6–8) [46]. After 6 and 24 h, the blue:yellow ratio was significantly elevated in PA-treated MmPTECs compared to BSA-treated cells (Figure 6A). In PA-treated cells, this parameter was not further affected by bafilomycin A_1_. In addition, PA-treated cells stained with acridine orange dye, which accumulates in lysosomes after protonation in this compartment [47], showed significantly less red fluorescence after 6 h. It is consistent with elevated lysosomal pH in these cells (Figure 6B,C). These observations indicated that PA impairs the lysosomal acidification from 6 h and thus likely the lysosomal function in MmPTECs. Because AMPK activators protected against the inhibition of the autophagic flux in PA-treated cells, their effect on lysosomal pH was assessed. After 6 (Figure 6D) or 24 h (Figure 6E), the pharmacological activation of AMPK in PA-treated cells was associated with a normalization of the LysoSensor^TM^ blue:yellow ratio. Bafilomycin A_1_ significantly increased this fluorescence ratio in these conditions (Figure 6D,E). Overall, AMPK activation protects PA-treated MmPTECs from autophagosome accumulation by preventing defective lysosomal acidification which maintains their degradation.

*p ≤ 0.05; **p ≤ 0.01; ***p ≤ 0.001; #p ≤ 0.05; ##p ≤ 0.01; ###p ≤ 0.001 between groups.

### PA alters lysosomal membrane permeability in MmPTECs which is prevented by AMPK activation

**Figure 7.**
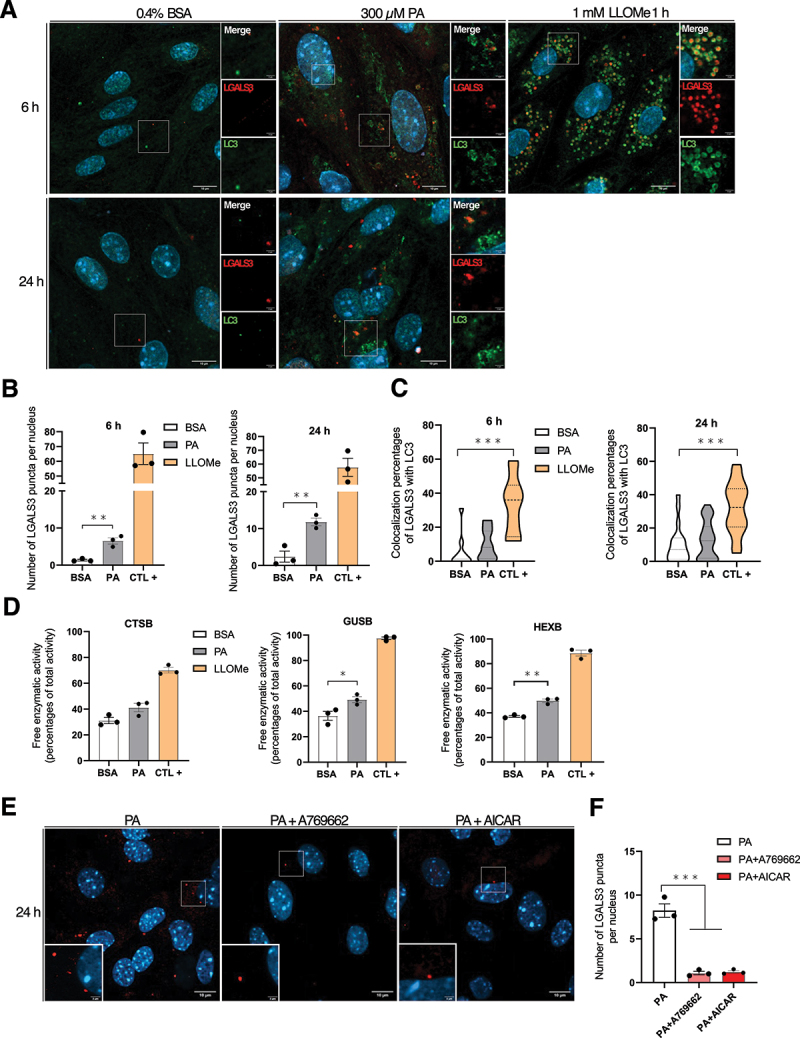
PA alters lysosomal membrane permeability in MmPTECs while pharmacological AMPK activation is protective. (A) Representative micrographs of cells treated for 6 or 24 h with 300 µM PA, 0.4% BSA or with 1 mM LLOMe for 1 h (positive control), fixed and immuno-stained for LGALS3 (red) and LC3 (green). (B, C) Quantifications of (B) the number of LGALS3 puncta per nucleus and (C) their percentages of colocalization with LC3 on 30 cells per group. (D) Free enzymatic activities of CTSB, GUSB and HEXB (expressed as percentages of total activities) in MmPTECs treated for 24 h with 300 µM PA, 0.4% BSA or with 1 mM LLOMe for 1 h (positive control). (E) Representative micrographs of cells treated for 24 h with 300 µM PA in the presence of 100 µM A769662 or 2 mM AICAR, fixed and immuno-stained for LGALS3 (red). (F) Quantifications of the number of LGALS3 puncta per nucleus on 30 cells per group. Data are presented as (B, D, F) means ± SEM or as (C) means and quarters of three independent biological experiments. Statistical analyses were performed by (B, D) Student’s unpaired t-test (comparison between BSA and PA only) or by (C, F) one-way ANOVA followed by Dunnett’s post-hoc test.

As we previously showed that lysosomal alkalinization occurs upon PA treatment, we next investigated lysosomal membrane permeabilization (LMP) in PA-treated cells, as well as pathways of lysosomal quality control. Depending on the intensity and the duration of the stress, different pathways might be initiated [30]. For instance, upon a stress that induces LMP, intraluminal glycans become accessible and are detected by LGALS3 (galectin 3), which recruits components of an autophagy initiation machinery to mediate lysophagy [48]. LMP is of particular interest to our study as this event is associated with disruption of the lysosomal proton gradient. After 6 and 24 h, PA-treated cells displayed significant higher number of LGALS3-positive puncta compared to BSA-treated cells but to lesser extent than cells treated for 1 h with 1 mM of the lysosomal-membrane damaging agent L-leucyl-L-leucine methyl ester hydrobromide (LLOMe) (Figure 7A,B). However, damaged lysosomes were unlikely engaged in lysophagy in PA-treated cells because the colocalization percentages of LGALS3 with LC3 were similar between BSA- and PA-treated cells after 6 or 24 h (Figure 7A,C). Besides, there was a significant increase in the colocalization percentage in cells treated with LLOMe as expected [49]. A consequence of LMP is the leakage of lysosomal enzymes into the cytosol, where they may trigger apoptosis and other cell death pathways [30]. Consistent with the elevated number of LGALS3 puncta in PA-treated cells, incubation of homogenates with the fluorogenic substrates of either GUSB (glucuronidase beta) or HEXB (hexosaminidase subunit beta) – two lysosomal acid hydrolases – revealed an increase of their free activities 24 h after treatment with PA (Figure 7D). Overall, these data showed that LMP occurs in MmPTECs upon PA-induced lipotoxicity, but it does not appear to induce lysophagy nor to modulate the viability of these cells (Figure S1E,F). Given that AMPK activators protect PA-treated cells from lysosomal alkalinization, we next wondered about their potential benefits on LMP. In MmPTECs treated with PA, AMPK activation mediated by A769662 or AICAR significantly suppressed LGALS3 detection after 24 h and thus prevented LMP (Figure 7E,F).

*p ≤ 0.05; **p ≤ 0.01; ***p ≤ 0.001 between groups.

### Lysosomal biogenesis is activated in response to PA-induced lysosomal damage in MmPTECs

**Figure 8.**
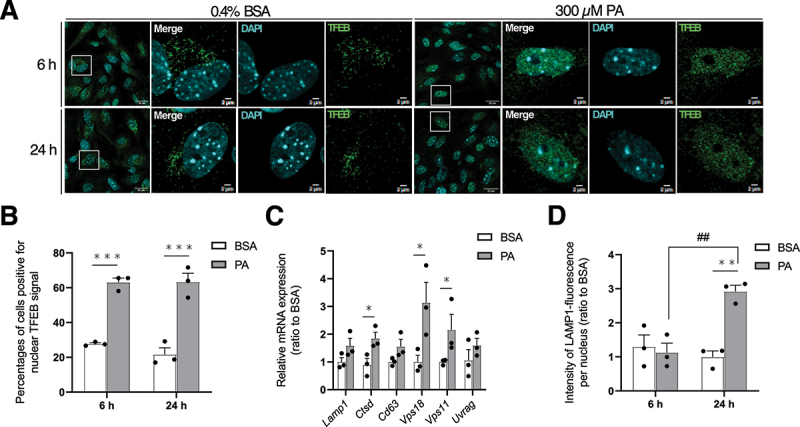
Lysosomal biogenesis is activated in response to pa-induced lysosomal stress in MmPTECs. (A) Representative micrographs of cells treated with 300 µM PA or 0.4% BSA for 6 or 24 h, fixed and immuno-stained for TFEB (green). (B) Quantification of the percentages of cells showing nuclear TFEB staining on more than 100 cells per group. (C) Relative mRNA expression of tfeb-targeted genes on MmPTECs treated for 24 h with 300 µM PA or 0.4% BSA. (D) Quantification of LAMP1-fluorescence intensities per nucleus by the integrated density tool on 30 cells per group (micrographs in Figure 5C). Data are presented as means ± SEM of three independent biological experiments. Statistical analyses were performed by (C) Student’s unpaired t-test, or two-way ANOVA followed by (B) Sidak’s or (D) Tukey’s post-hoc test.

Lysosomal biogenesis is another pathway of lysosomal quality control which aim to replace the pool of damaged organelles [30]. Interestingly, upregulation of lysosomal gene expression has been shown in kidneys of obese male mice [18]. We were thus interested to test whether it occurred following lipid stress in MmPTECs as well as to identify the molecular actors involved. To do so, the subcellular localization of TFEB (transcription factor EB), which regulates the expression of genes encoding the coordinated lysosomal expression and regulation/CLEAR network [50], was assessed in PA-treated MmPTECs. TFEB staining showed nuclear signal in PA-treated cells for 6 or 24 h while it displayed a cytosolic localization in BSA-treated cells (Figure 8A,B). PA-treated cells after 24 h showed significant upregulation of some TFEB-targeted genes including *Ctsd* (cathepsin D), *Vps11* (VPS11 core subunit of CORVET and HOPS complexes) and *Vps18* (Figure 8C, 6 h data shown in Figure S7A). It correlated with the stronger fluorescent signal for LAMP1 observed in PA-treated cells after 24 h (Figure 8D, micrographs in Figure 5C). Interestingly, TFEB nuclear localization and expression of target genes were not modified by the addition of AMPK activators in PA-treated cells (Figure S8). These findings are not unexpected (given that AMPK promotes lysosomal biogenesis [28]) and suggest that the effect of PA on TFEB is independent of AMPK inhibition. Overall, these data demonstrated an induction of lysosomal biogenesis upon lipid challenge in MmPTECs which is likely mediated by TFEB.

*p ≤ 0.05; **p ≤ 0.01; ***p ≤ 0.001 *versus* corresponding BSA group; ##p ≤ 0.01 between PA groups.

### The PA-induced lysosomal dysfunction drives MmPTEC dedifferentiation while AMPK activation is protective

**Figure 9.**
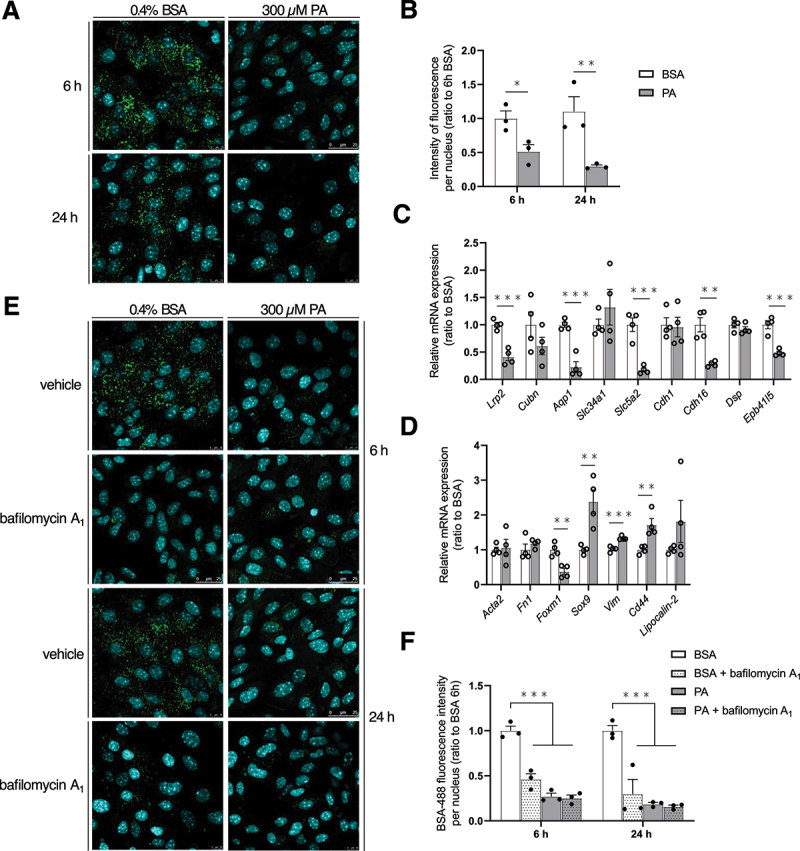
PA-induced lysosomal dysfunction drives MmPTEC dedifferentiation. (A) Representative micrographs of MmPTECs treated with 300 µM PA or 0.4% BSA for 6 or 24 h, incubated with 100 µg/mL BSA-488 for 30 min at 37°C and fixed. (B) Quantification of BSA fluorescence intensities per nucleus by the integrated density tool on more than 100 cells per group. (C, D) Relative mRNA expression of (C) differentiation and (D) dedifferentiation markers on MmPTECs treated with 300 µM PA or 0.4% BSA for 24 h. (E) Representative micrographs of MmPTECs treated for 6 or 24 h with 300 µM PA or 0.4% BSA with or without 2 nM bafilomycin A_1_ for last 6 h, incubated with 100 µg/mL BSA-488 for 30 min at 37°C and fixed. (F) Quantification of BSA fluorescence intensities per nucleus by the integrated density tool on more than 100 cells per group. Data are presented as means ± SEM in three or four independent biological experiments as indicated on the charts. Statistical analyses were performed by (C, D) Student’s unpaired t-test or by (B, F) two-way ANOVA followed by Dunnett’s post-hoc test.

Despite the emergence of tubulocentric view in metabolic CKD, the effects of lipotoxicity on PTEC differentiation phenotype remain largely unknown. It is likely explained by the inability of main *in vitro* models to recapitulate *in vivo* PTEC features such as polarity, transporter expression and receptor-mediated endocytosis [22]. Here, we showed that PA-treated cells for 6 or 24 h displayed significantly less cytosolic fluorescence intensity after BSA-488 uptake (Figure 9A,B). It translated alterations in protein reabsorption secondary to lipid overload in MmPTECs and thus cell dysfunction. After 24 h of PA, cells showed significantly decreased expression of differentiation markers including *Lrp2/megalin* (LDL receptor related protein 2), the receptor involved in protein endocytosis, transporters (*Aqp1* [aquaporin 1 (Colton blood group)] and *Slc5a2* [solute carrier family 5 member 2]) as well as genes encoding proteins of tight junctions and desmosomes (*Cdh16* [cadherin 16] and *Epb41l5* [erythrocyte membrane protein band 4.1 like 5]) (Figure 9C). It was concomitant with an upregulation of some injury and mesenchymal markers (*Sox9* [SRY-box transcription factor 9], *Vim* [vimentin] and *Cd44*) (Figure 9D). No change in the expression of these markers was observed after 6 h of PA treatment (Figure S7B,C). These results suggested that PA alters the differentiation phenotype of MmPTECs and that endocytic alterations occur prior to the modifications in gene expressions. Protein endocytosis relies on the availability of receptors at the apical site, effective endocytosis and intracellular trafficking for ligands/proteins to be degraded in lysosomes [51]. We thus used bafilomycin A_1_ to determine whether impaired lysosomal acidification by itself might modify this process. Control cells treated with bafilomycin A_1_ for 6 h displayed a significant decrease in the uptake of BSA-488 (Figure 9E,F). Again, this suggested an impairment in protein reabsorption and underlined the need for effective lysosomal function in this endocytic pathway. The insensitivity of PA-treated cells to bafilomycin A_1_ clearly demonstrated the contribution of lysosomal dysfunction on the phenotype of impaired endocytosis in PA-treated MmPTECs.

*p ≤ 0.05; **p ≤ 0.01; ***p ≤ 0.001 *versus* corresponding BSA group.

**Figure 10.**
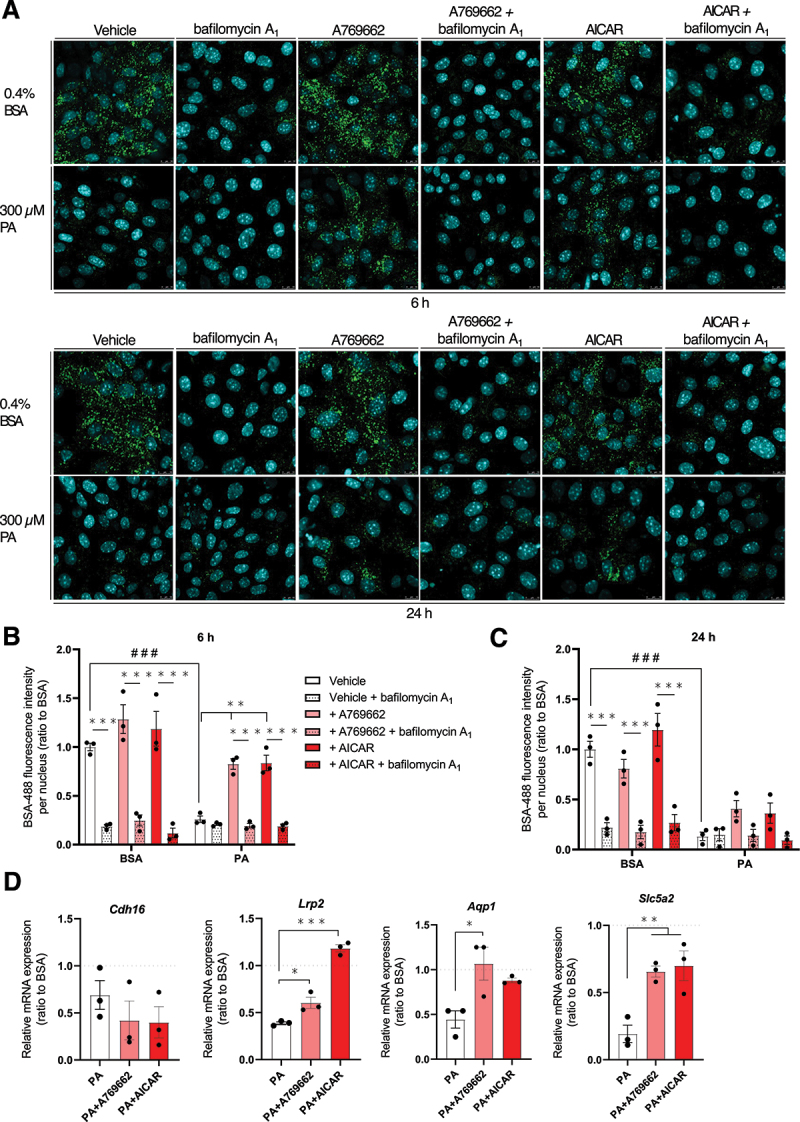
AMPK activation protects MmPTECs from pa-induced dedifferentiation. (A) Representative micrographs of cells treated for 6 or 24 h with 300 µM PA or 0.4% BSA in the presence of 100 µM A769662 or 2 mM AICAR with and without 2 nM bafilomycin A_1_ for last 6 h, incubated with 100 µg/mL BSA-488 for 30 min at 37°C and fixed. (B, C) Quantification of BSA fluorescence intensities per nucleus by the integrated density tool after (B) 6 and (C) 24 h on more than 100 cells per group. (D) Relative mRNA expression of differentiation markers (*Cdh16*, *Lrp2*, *Aqp1* and *Slc5a2*) on MmPTECs treated for 24 h with 300 µM PA in the presence of 100 µM A769662 or 2 mM AICAR. Data are presented as means ± SEM in three independent biological experiments. Statistical analyses were performed by (D) one-way ANOVA followed by Dunnett’s post-hoc test or by (B, C) two-way ANOVA followed by Tukey’s post-hoc test.

Finally, as we showed that AMPK activators protected from lysosomal dysfunction in PA-treated cells (Figure 6), their potential in the maintenance of protein endocytosis was assessed. AMPK activation in PA-treated cells significantly increased the cytosolic BSA-488-related fluorescence (Figure 10A,B). This protection was alleviated by bafilomycin A_1_ which strengthened the importance of lysosomal homeostasis. The protective effect of AMPK activators on protein endocytosis was, however, relatively transient after 24 h (Figure 10A,C). However, the benefits of AMPK activation to counteract the deleterious effect of PA on MmPTEC dedifferentiation after 24 h were highlighted. Indeed, the addition of A769662 or AICAR significantly increased the expression of differentiation markers such as *Lrp2*, *Aqp1* and *Slc5a2* in PA-treated cells after 24 h (Figure 10D).

*p ≤ 0.05; **p ≤ 0.01; ***p ≤ 0.001 in BSA or PA treatment groups; ###p ≤ 0.001 between treatment groups.

## Discussion

Although the impact of lipotoxicity on autophagic flux has been studied in PTECs, conflicting results and interpretations remained prior to our study. Recent studies on non-renal cells show that autophagic changes secondary to FA excess result from direct lysosomal injury. In this study, we placed lysosomes at the cornerstone of PTECs physiology and lipotoxic phenotype. Indeed, impaired acidification was shown in PTECs exposed to PA, subsequently leading to the accumulation of autophagosomes and cell dedifferentiation. We also confirmed the protection of AMPK activation against lipotoxicity previously demonstrated by our group [13] and brought the underlying molecular mechanisms. In PTECs, AMPK prevented PA-induced defective lysosomal acidification, autophagosomes accumulation, and cell dedifferentiation.

One of the main findings of the present study is that saturated FAs such as PA impaired lysosomal acidification in PTECs. Autophagy did not seem to be induced upon lipid challenge, which is in accordance with decreased AMPK activity and MTORC1 activation. Elevated number of autophagosomes observed in PA-treated cells thus mainly resulted from an absence of lysosomal degradation. After refuting the hypotheses of mitophagy and lipophagy, we showed that autophagy targeted Ub-tagged aggregates in response to PA which are ultimately found in lysosomes. The lysosomal dysfunction highlighted in the present work is of physiological relevance knowing that lysosome enlargement and accumulation were described in proximal tubules of obese male mice [13,18]. Notably, we showed that lysosomal dysfunction is an early event of lipotoxicity as it occurred as soon as 6 h after PA exposure. At that time point, the mitochondrial network was not modified, and LDs were barely detectable. Several mechanisms have been proposed to explain FA-mediated lysosomal alkalinization. While lysosomal calcium homeostasis alterations have been proposed to play a role [25,52], several studies directly demonstrated the involvement of the lysosomal V-ATPase [53]. An inhibition of this pump has been shown upon lipotoxicity due to excessive disassembly of the soluble hydrolytic V_1_ domain from the membrane-associated V_0_ as a result of its palmitoylation [54,55] or oxidative denaturation [26]. The relevance of these mechanisms in our experimental conditions remains to be determined. Lysosomal alkalinization was concomitant with LMP in PTECs exposed to PA; both are tightly interconnected as the rupture of lysosomal membranes dissipates the proton gradient. Several studies have shown that the disruption of lysosomal pH in control conditions by lysosomotropic drugs is not sufficient to trigger LMP despite causing lysosomal enlargement and inhibition of degradative capacity [56–58]. On the one hand, it suggests that additional mechanisms occur and alter the permeability of lysosomal membranes in PA-treated cells. On the other hand, we showed that lysosomes are highly solicited in conditions of lipotoxicity in PTECs, their alkalinization leads to accumulation of substrates at higher rates (notably Ub aggregates) which may induce LMP due to excessive expansion. Finally, because many degradative pathways converge to lysosomes, their alkalinization exacerbate cell function decline secondary to lipotoxicity. It is perfectly illustrated by studies using lysosomal acidifying nanoparticles. Upon lipid challenge, lysosomal delivery of protons restores cathepsin L activity, autophagic flux but also cell homeostasis and function in cultured hepatocytes and β-cells [59]. One step further, acidifying nanoparticles injected in HFD-fed animals reverse dyslipidemia, hyperglycemia, hepatic steatosis and inflammation [60]. In this regard, we pointed out AMPK as another valuable target to protect lysosomes against the deleterious effect of lipotoxicity.

AMPK activity has been previously shown to be impaired in the kidneys of mice [15,16] and patients [17] with metabolic CKD. The present study demonstrated the direct effect of PA on the diminution of AMPK activity in PTECs, likely as part of a nutrient stress response. Indeed, PA uptake is associated with higher β-oxidation rates, reduced AMP:ATP and higher NADH production [1]. Emerging evidence also describes a role for lysosomes in the regulation of energy-sensor pathways. Starvation is sensed by the lysosomal V-ATPase-Ragulator complex which facilitates AMP-dependent STK11/LKB1-mediated AMPK activation [27]. In conditions of high energy, STK11 is sequestered by AXIN in the cytosol preventing AMPK activation while the V-ATPase-Ragulator complex activates MTORC1. This signaling pathway relies on precise regulations, and we might extrapolate that the loss of acidification observed secondary to PA in PTECs contributes to AMPK and MTORC1 activity changes. It is reinforced by evidence showing MTORC1 sequestration to lysosomes and hyperactivation in various lysosomal dysfunction and lysosomal storage disorders (LSDs) [61–63].

AMPK activation, either by AICAR or A769662, protected PTECs against the defective lysosomal acidification observed upon lipotoxicity. Restoration of lysosomal pH strongly reduced the number of autophagosomes, Ub aggregates, and prevented LMP in PA-treated cells. As expected, AMPK activation was associated with stronger phosphorylation of ACAC, but no change was observed regarding the phosphorylation of ULK1, and the reason behind remains to be determined. However, this finding supports our conclusion that autophagosome accumulation mainly resulted from defective lysosomal degradation. The AMPK protection against the deleterious effect of lipotoxicity on lysosomes is also in line with our previous study showing that AICAR administration is associated with reduced lysosomal enlargement in proximal tubules of mice with obesity-induced CKD [13]. AMPK was found to be necessary for basal lysosomal function. The suppression of the two AMPK catalytic units (*prkaa1* [protein kinase AMP-activated catalytic subunit alpha 1] *prkaa2* double knockout) in mouse embryonic fibroblasts leads to defective lysosomal catalytic activity, loss of acidification and elevated calcium concentration [29]. Mechanistically, how AMPK pharmacological activation prevents PA-induced defective lysosomal acidification remains to be determined. The kinase activates PIKFYVE (phosphoinositide kinase, FYVE-type zinc finger containing) which catalyzes the phosphatidylinositol-3,5-bisphosphate synthesis needed for proper activity of MCOLN1 (mucolipin TRP cation channel 1) and lysosomal homeostasis [64]. Suppression of this pathway due to AMPK or PIKFYVE inhibition is associated with lysosomal dysfunction [65]. Additionally, AMPK has been shown to promote the two V-ATPase subunit assembly and subsequently the pumping activity in HEK293T cells [66]. These pathways provide hypotheses to decipher the underlying events behind AMPK protection in PA-treated PTECs which will be assessed in future studies.

The importance of lysosomal quality control in PTECs has been recently shown with the activation of TFEB-mediated biogenesis and lysophagy in these cells of mice suffering from crystal nephropathy [67]. Injured lysosomes are observed in all renal tubular cells in the disease, but these pathways are only activated in proximal tubules. The present work characterized how these pathways are affected in PTECs in response to lysosomal stress due to lipotoxicity. First, the engagement of damaged organelles in lysophagy was not observed, and the reason behind the absence of recruitment of core autophagy regulators in response to LGALS3 remains to be determined. However, it raises the question about the fate of these altered organelles. Interestingly, no cell death was observed in PA-treated cells despite evidence of LMP. Lysosomal exocytosis is an unconventional secretion process where lysosomes translocated from perinuclear area to fuse with plasma membrane thus releasing their content in the extracellular space [68]. This pathway was shown to be stimulated upon TFEB activation [69], and activated in renal cells in a model of LSD [70] and is thus interesting to study in PTECs upon lipid challenge. In contrast to lysophagy, TFEB-mediated lysosomal biogenesis was highlighted in PA-treated PTECs which likely aim to replace the pool of dysfunctional organelles. It is in line with data of our group showing enhanced expression of lysosomal genes in kidneys of obese male mice [18] and with the observation of TFEB translocation in macrophages of obese mice and *in vitro* upon PA treatment [52]. The present work showed that after 24 h, new lysosomes were formed in PA-treated PTEC as evidenced by increased mRNA expression and LAMP1 protein abundance, but pH ratio indicated neutral lysosomal pH at that time, suggesting that these lysosomes did not display acidic pH and were thus likely unfunctional.

In view of the inability of the biogenesis to restore lysosomal acidification secondary to PA, we wondered about the effect on PTEC function. We showed that PA exposure quickly altered protein endocytosis, which is a direct consequence of lysosomal alkalinization, as bafilomycin A_1_ itself recapitulated the phenotype. Other factors might nevertheless contribute because PA-treated PTECs displayed a down regulation of *Cubn* and *Lrp2* after 24 h, so receptors might become limiting. Reduced protein endocytosis might be seen as a protective mechanism to avoid putting additional burden on ineffective lysosome and/or might be a manifestation of a broader event such as PTEC fate change. Indeed, epithelial cells might undergo dedifferentiation characterized by the loss of apico-basal polarity markers [71]. Alterations of gene expression that determine the PTEC profile were highlighted secondary to PA with reduced expression of *Lrp2, Aqp1, Slc5a2, Cdh16* and *Epb41l5* after 24 h. Interestingly, impaired protein endocytosis and some of these expression changes are reported in LSD (genetic *Ctns* [cystinosin, lysosomal cystine transporter] deletion) on cultured PTECs and validated in mice [63]. This disease is associated with MTORC1 hyperactivation while rapamycin reverses PTEC dedifferentiation. It is in line with data of this study showing that AMPK activation prevented PA-induced defective protein reabsorption (by maintaining lysosomal acidification) and preserved differentiation marker expression such as *Lrp2*, *Aqp1* and *Slc5a2*. Epithelial fate changes have long been associated with external factors including pro-inflammatory mediators [72]. In accordance with literature, we showed that lysosomal dysfunction is a kind of injury able to modulate epithelial cell destiny. Even if the underlying mechanisms are far for being understood, AMPK and MTORC1 dysregulations should be considered in this context.

Proteinuria refers to elevated and persistent proteins in urines and might be secondary to glomerular filtration barrier alterations and/or defective reabsorption by PTECs [73]. In metabolic CKD, proteinuria is told to result from filtration barrier permeabilization. Protein leakage thus increases the reabsorption burden and shear stress on PTECs, which will activate compensatory mechanism at first, while it finally mediates injury [74]. However, our results highlighted the direct effect of dysregulated lipid metabolism on these cells. Loss of differentiation markers in tubular proteinuria were observed in obesity and diabetic CKD animal models [7,75]. These two features were recapitulated in this work in PTECs upon increased uptake of saturated FAs. It reinforces the tubulocentric view which postulate the crucial role of tubulopathy upon metabolic stress [76]. Another evidence supporting this concept is the demonstration that lipotoxicity of PTECs is associated with injury marker upregulation such as *Sox9* and *Cd44*. Even if SOX9 expression is needed for repair after acute injury [77], elevated SOX9 was found in CKD biopsies and correlated with the severity of tubulointerstitial fibrosis and tubular cell damage [78]. In the same line, CD44 is expressed in renal tubules during maladaptive repair and interstitial fibrosis [79]. In addition, the significant upregulation of *Vim* in response to PA, in parallel to loss of epithelial markers, translates epithelial-to-mesenchymal transition (EMT) [80]. EMT is of great importance in renal diseases as it provides one third of fibroblasts in conditions of tubulo-interstitial fibrosis [71], a feature commonly associated with end-stage renal disease. In obesity-induced CKD, low-grade interstitial fibrosis is commonly reported but the underlying mechanisms remain to be understood. Because we showed that PTECs undergo EMT, they might play a role upon metabolic injury in the occurrence of tubulo-interstitial fibrosis, which need to be further addressed.

In conclusion, our data highlighted the importance of lysosomal homeostasis for PTEC physiology. Increasing recent evidence on several cell types point out lysosomes as direct target of injury upon excess of saturated FAs. The present study reinforced this hypothesis; lysosomal acidification was shown in response to PA which led to loss of both PTEC function and differentiation profile. In addition, we highlighted the potential of lysosomal function maintenance as a valuable therapeutic strategy to counteract the effect of lipotoxicity in PTECs and further demonstrated the benefits of AMPK activation in this context.

## Material and methods

### Antibodies

The following antibodies were used for immunofluorescence (IF) or western blot analysis (WB): EIF4EBP1 (Cell Signaling Technology, 9644S; WB, 1:1000); phospho-EIF4EBP1 (Thr37/46; Cell Signaling Technology, 2855S; WB, 1:1000); ACAC (Cell Signaling Technology, 3676; WB, 1:1000); phospho-ACAC (Ser79; Cell Signaling Technology 11,818; WB, 1:100); PRKAA/AMPKα (Cell Signaling Technology, 2532; WB, 1:1000); phospho-PRKAA/AMPKα (Thr172; Cell Signaling Technology, 2535; WB, 1:1000); ACTB (Sigma-Aldrich, A5441; WB, 1:5000); cleaved-CASP3 (Cell Signaling Technology, 9661; WB, 1:1000); LGALS3 (Santa Cruz Biotechnology, sc -23,938; IF, 1:100); LAMP1 (Developmental Studies Hybridoma Bank, 1D4B-C; IF, 1:100); LAMP2 (Developmental Studies Hybridoma Bank, ABL93; IF, 1:100); LC3 (Sigma-Aldrich, L7543; WB, 1:1000; IF, 1:100); OXPHOS cocktail, (abcam, ab110413; IF, 1:500); PLIN2 (Progen, GP40; IF, 1:100); SQSTM1 (Cell Signaling Technology, 23214S; WB, 1:1000; IF, 1:100); RPS6KB (Cell Signaling Technology, 2708; WB, 1:1000); phospho-RPS6KB (Thr389; Cell Signaling Technology, 9234; WB, 1:1000); TFEB (Bethyl Laboratories, A303-673A; IF, 1:100); ubiquitin (Santa Cruz Biotechnology, sc -166,553; WB, 1:1000; IF, 1:100); ULK1 (Cell Signaling Technology, 8054; WB, 1:1000) and phospho-ULK1 (Ser555; Cell Signaling Technology, 5869; WB, 1:1000).

### Reagents

When indicated and for referred incubation times, cell culture media were supplemented with: 100 µM A769662 (Sigma-Aldrich, SML2578); 2 mM 5-aminoimidazole-4-carboxamide-1-beta-D-ribofurasonide (AICAR; Toronto Research Chemicals, A611700); 2 nM bafilomycin A_1_ (Sigma-Aldrich, B1793); 10 µM camptothecin (Merck, PHL89593); 10 µM of carbonyl cyanide 4-(trifluoromethoxy)phenylhydrazone (FCCP, MedChemExpress, HY-100410); 1 mM lysosomal-membrane damaging agent L-leucyl-L-leucine methyl ester hydrobromide (LLOMe, Sigma-Aldrich, L7393); 10 µM N-benzyloxycarbonyl-L-leucyl-L-leucyl-L-leucinal (MG132; Sigma-Aldrich, M7449) or 300 µM oleate (OA; Cayman Chemical 29,557).

### Primary MmPTEC isolation and culture

The study conformed to the APS Guidelines for the Care and Use of Animals and was approved by the Animal Ethics Committee of the University of Namur. The kidneys were harvested from 4- to 6-week-old wild type C57Bl/6J mice and proximal tubule (PT) segments were recovered as previously reported [63,81]. Fresh PT were seeded in 15 µg/mL FN1 (fibronectin 1)-coated (R&D Systems, 1030-FN) 6-well plates and cultured at 37°C in a humid atmosphere containing 5% CO_2_ in medium composed of DMEM/F12 (Thermo Fisher Scientific 21,041–025) supplemented with 5% (vol:vol) heat-inactivated fetal bovine serum (HIS; Dulis, 500105N1N), 15 mM HEPES (Thermo Fisher Scientific 15,630,056), 0.55 mM sodium pyruvate (Sigma Aldrich, P2256), 0.1 ml/L non-essential amino acids (Sigma Aldrich, M7145), hydrocortisone, human EGF, epinephrine, insulin, triiodothyronine, transferrin, and gentamicin+amphotericin (SingleQuots® kit; Lonza, CC-4127), pH 7.4, 325 mOsm/kg H_2_O. After 48 h, the seeding medium was replaced by fresh cell culture media with 2.5% HIS. After 7 days, when cultured cells organized as a confluent monolayer of MmPTECs, they were used for further analysis. These cells were negatively tested for mycoplasma contamination.

Sodium palmitate (Sigma-Aldrich, P9767) was added in 10% free-FA BSA (Sigma-Aldrich, A8806) solution at 37°C under agitation to reach PA concentration of 7.5 mM stock solution and a PA:BSA molar ratio of 5:2. The concentration was assessed after each complexation using the non-esterified FA assay kit (Fujifilm WAKO, 434–91795 and 436–91995,). MmPTECs were treated with 300 µM PA or 0.4% BSA as a vehicle.

### Fluorescence microscopy

#### Lipid droplet imaging

Cells were incubated for 15 min at 37°C with 2.5 µM BODIPY493/503 (Thermo Fisher Scientific, D3922) and 1 µM DAPI (Thermo Fisher Scientific, D1306). After two PBS (Dulis, L0616–500) washes, living cells were imaged with a Leica TCSSP5 II confocal laser-scanning microscope (Leica Microsystems, Belgium). Quantifications of LD size and number were performed using FIJI v.2.1.0. and the “*MRI Lipid Droplets Tool*” plugin (Figure 1B,C, S1B and S1C).

#### Lumenal lysosomal and autophagosomal pH assessment

Cells were transduced with Premo™ autophagy tandem sensor RFP-GFP-LC3B kit (Thermo Fisher Scientific, P36239) and treated 24 h later. Cells were then incubated for 5 min with 1 µM DAPI and fixed with ice-cold methanol:acetone (1:1) (Carl Roth, 7342.1 and T161.1, respectively) for 10 min and mounted in Fluoromount G (Thermo Fisher Scientific, 00-4958-02). Samples were then imaged with a Zeiss LSM 900 confocal laser-scanning microscope equipped with an Airyscan 2 multiplex system (Carl Zeiss, Germany). The number of acidic LC3 puncta was determined by counting the GFP^−^ RFP^+^ puncta while the number of LC3 structures at neutral pH was obtained by the number of GFP^+^ RFP^+^ LC3 puncta per cell (Figures 1H, 1I, 3E, 3F, S4E and S4F).

For lysosomal pH assessment, cells were seeded on 96-well black with clear flat plate (Corning 353,219). After treatment, cells were incubated with 5 µM LysoSensor Yellow/Blue DND-160 (Thermo Fisher Scientific, L7545) for 3 min at 37°C. After two PBS washes, fluorescence emissions at 440 (lysosomal pH of 6–8) and 540 nm (lysosomal pH of 3–5) were measured with SpectraMax i3× Multi-Mode Microplate Detection Platform (Molecular Devices, United States). Lysosomal pH was also assessed through cell incubation with 2.5 µM Acridine Orange (Sigma-Aldrich, A-6014) for 15 min at 37°C. After two PBS washes, living cells were imaged with a Zeiss LSM 900 confocal laser-scanning microscope. The red:green fluorescence ratios were quantified by the Integrated Density tool on FIJI v.2.1.0. software (Figure 6C).

#### Bsa-upatke endocytic assay

The protein endocytic uptake was monitored in MmPTECs following incubation for 30 min at 37°C with 100 μg/mL BSA-Alexa Fluor 488 (Thermo Fisher Scientific, A13100) in DMEM/F12 (Thermo Fisher Scientific 21,041–025). After an acid wash, cells were rinsed and then fixed with 4% paraformaldehyde (VWR Chemicals, MFCD00133991) for 10 min and mounted with Fluoromount G. A Leica TCSSP5 II confocal laser-scanning microscope was then used to measure BSA-Alexa Fluor 488 endocytosis. Fluorescence intensities were quantified by the Integrated Density tool on FIJI v.2.1.0. software (Figures 9A, 9E, 10A).

#### Sample preparation and analyses of immunofluorescence

After fixation with 4% paraformaldehyde for 10 min, cells were incubated for 30 min in a permeabilization and blocking buffer composed of 15 mM glycine (Carl Roth, 3908.2), 0.05% (wt:vol) saponin (Sigma-Aldrich 47,036), 0.5% (wt:vol) BSA (Carl Roth, 1ET6.3), 50 mM NH_4_Cl (Sigma-Aldrich, A9734), pH 7.4. For LC3 immunostaining, cells were fixed with ice-cold methanol:acetone (1:1) for 10 min. Cells were then incubated with primary antibodies overnight at 4°C, rinsed three times with blocking buffer and incubated with fluorophore-conjugated Alexa Fluor secondary antibodies (1:400; Thermo Fisher Scientific, A32790, A10042, A32766, A11031, A11075, A11077) and 1 µM DAPI for 1 h and finally mounted with Fluoromount G. Samples were imaged with a Zeiss LSM 900 confocal laser-scanning microscope equipped with an Airyscan 2 multiplex system (Figures 4A, 4F, 5A, 5C, 6B, 7A, 7E, S5A, S5B and S6A) or Leica TCSSP5 II confocal laser-scanning microscope (Figure 8A and S8A).

Fluorescent micrographs were quantified using the FIJI v.2.1.0 software. Colocalization studies were assessed on individual cells with the JACOP plugin using Mander’s coefficients (Figures 4C, 5B, 7C, S5A, S5C, S5D and S6D) or an objects-based method (Figure 4H). In case of LC3 immuno-staining, a threshold was applied first to keep punctuated structures (indicative of autophagosomes) and remove diffuse cytosolic signal (Figures 5A, 7A and S5A,B). The percentages of cells with TFEB nuclear localization were obtained by calculating the ratio between the number of cells with positive nuclear TFEB-related fluorescence and the total number of cells (Figure 8B). The number of ubiquitin (Figures 4B, 4G and 5D) and LGALS3 (Figure 7B,F) puncta were calculated with the “*Analyze Particles*” function (for ub puncta, only those higher than 0.5 µm^2^ were kept) and normalized by the number of nucleus.

### Western blot analyses

Cells were lysed in 100 µL/well of ice-cold lysis buffer composed of cell lysis buffer (1:10) (Cell Signaling Technology, 9803) and halt protease and phosphatase inhibitor cocktail (1:100; Thermo Fisher Scientific 87,786). An equivalent of 20 µg of proteins were loaded on a bis-Tris 4–12% or a Tris-Acetate 3–8% gel (Thermo Fisher Scientific, NP0323 or EA03755, respectively) and separated with 200 V for 45 min. Proteins were then transferred on a polyvinylidene fluoride membrane (Sigma-Aldrich, IPFL00010) which were blocked with Intercept Tris Buffer Saline (TBS) buffer for 1 h at room temperature (Li-Cor Biosciences, 927–60001) and incubated overnight at 4°C with primary antibodies. The next day, membranes were rinsed with TBS containing 0.1% Tween 20 (Carl Roth, 9127.1) and incubated for 1 h with secondary antibodies (1:10000; Li-Cor Biosciences). Fluorescence was quantified with Odyssey LI-COR scanner (LI-COR Biosciences, United States).

### AMPK immunoprecipitation and assay

AMPK phosphotransferase activity was measured as previously described [82]. Briefly, total AMPK was immunoprecipitated from 50 µg of cell lysates with Protein G-Sepharose 4 Fast Flow beads (Cytivia, 17-0618-01) coupled to 2 µg of anti-PRKAA1/AMPKα1 and 2 µg of anti-PRKAA2/AMPKα2 antibodies. After several washes, AMPK activity was measured for 45 min at 30°C in a reaction mixture containing 50 mM HEPES, pH 7.4, 10 mM MgCl_2_, 100 µM of AMARA peptide (homemade, gift from the CARD, UCLouvain), 100 µM of ATP (Roche Applied Science 06,529,194,103) in the presence of [γ-^32^P] ATP (1 µCi; Revvity, BLU002H). The reaction was terminated by transferring the reaction mixture onto P81 papers (homemade, gift from the CARD, UCLouvain) immersed in 75 mm orthophosphoric acid. After washing, the filter papers were dried, and ^32^P incorporation into the AMARA peptide was measured by Cherenkov counting in a scintillation counter Ti-carb 2810 TK (Perkin Elmer, United States). One milliunit (mU) was defined as the incorporation of 1 pmol ^32^P on the substrate per minute and per µg of proteins.

### Assessment of lysosomal enzyme free activities

The free activities of CTSB (cathepsin B), GUSB and HEXB were assessed as published [83]. Briefly, cells were washed twice with ice-cold 0.25 M sucrose (Sigma-Aldrich, S9378), collected after scraping in 300 µL sucrose and homogenized by six passages in a glass homogenizer (Kimble Kontes 9,651,630). The nuclear fraction was discarded after centrifugation at 1,200 g for 1 min 30 sec (TLX ultracentrifuge and TLA100.3 rotor, Beckman Coulter, United States). A volume of 10 µL supernatants were incubated at 37°C for 15 min with 5 mm 4-methylumbelliferyl-N-acethyl-B-D-glucosaminide (HEXB substrate; Glycosynth Limited 37,067-30-4), 5 mM 4-methylumbelliferyl-B-D-glucuronide (GUSB substrate; Sigma-Aldrich, M9130) or with 0.5 mM Z-Arg-Arg-7-amido-4-methylcoumarin hydrochloride (CTSB substrate; Merck, C5429). Substrates were previously diluted in 0.5 M sucrose and 100 mM citrate (dilution 1:1, pH 4.5) in the absence (free activity) or in the presence (total activity) of 0.05% Triton X-100 (Sigma-Aldrich, T9284). The reaction was stopped with 1 mL of buffer composed of 50 mM glycine, 5 mM EDTA and 0.5% Triton X-100 (pH 10.5) and fluorescence intensities were read by the Versafluor Fluorometer (Biorad, United States).

### RNA extraction and RT-qPCR

Total RNA was extracted using the ReliaPrep RNA Tissue Miniprep System (Promega, Z6111) following manufacturer’s instructions. RNA concentrations were assessed with NanoDrop 1000 (Thermo Fisher Scientific, United States). Reverse transcription was performed with the Transcriptor First Strand cDNA Synthesis Kit (Roche Applied Science 04,897,030,001) to convert 1 μg of RNA into cDNA. Quantitative PCR mixtures were composed of 10 µL of Takyon^TM^ ROX SYBRR Master Mix (Eurogentec, UF-RSMT-B0701), 2.5 µL of forward and reverse primers (final concentration of 300 nM) and 5 µL of 5 ng/µL cDNA per well. Primers (Table S1) (Integrated DNA Technologies) were previously analyzed for dissociation curves and melting temperatures. Amplification reactions were performed using the LightCycler® 96 System (Roche Applied Science, Germany). Relative gene expressions were calculated using the 2^−ΔΔCT^ method with *Actb* used as housekeeping gene.

### Statistical analyses

Results were presented as means ± standard error of the means (SEM). Differences between groups were evaluated using one or two-way analysis of variance (ANOVA) followed by different post-hoc tests depending on the application as indicated in Figure legends. Unpaired Student’s t-tests were used for comparison between two groups. The level of significance was defined as 0.05 and *p* values were indicated by symbols in the Figure legends. All experiments were performed at least three times or more (as indicated on the charts). GraphPad Prism software v. 8.0.2 (GraphPad Software) was used for statistical analyses and chart generation.

## Supplementary Material

revised supplemental R3.docx

## References

[1] Bhargava P, Schnellmann RG. Mitochondrial energetics in the kidney. Nat Rev Nephrol. 2017;13(10):629–646. doi: 10.1038/nrneph.2017.107 28804120 PMC5965678

[2] Thomas M, Schreiner G. Contribution of proteinuria to progressive renal injury: consequences of tubular uptake of fatty acid bearing albumin. Am J Nephrol. 1993;13(5):385–398. doi: 10.1159/000168653 8116691

[3] Khan S, Cabral PD, Schilling WP, et al. Kidney proximal tubule lipoapoptosis is regulated by fatty acid transporter-2 (FATP2). J Am Soc Nephrol. 2018;29(1):81–91. doi: 10.1681/ASN.2017030314 28993506 PMC5748912

[4] Yang X, Okamura DM, Lu X, et al. Cd36 in chronic kidney disease: novel insights and therapeutic opportunities. Nat Rev Nephrol. 2017;13(12):769–781. doi: 10.1038/nrneph.2017.126 28919632

[5] Zeni L, Norden AGW, Cancarini G, et al. A more tubulocentric view of diabetic kidney disease. J Nephrol. 2017;30(6):701–717. doi: 10.1007/s40620-017-0423-9 28840540 PMC5698396

[6] Thrailkill KM, Nimmo T, Bunn RC, et al. Microalbuminuria in type 1 diabetes is associated with enhanced excretion of the endocytic multiligand receptors megalin and cubilin. Diabetes Care. 2009;32(7):1266–1268. doi: 10.2337/dc09-0112 19366958 PMC2699744

[7] Tojo A, Onozato M, Ha H, et al. Reduced albumin reabsorption in the proximal tubule of early-stage diabetic rats. Histochem Cell Biol. 2001;116(3):269–276. doi: 10.1007/s004180100317 11685557

[8] Garofalo C, Borrelli S, Minutolo R, et al. A systematic review and meta-analysis suggests obesity predicts onset of chronic kidney disease in the general population. Kidney Int. 2017;91(5):1224–1235. doi: 10.1016/j.kint.2016.12.013 28187985

[9] Kang HM, Ahn SH, Choi P, et al. Defective fatty acid oxidation in renal tubular epithelial cells has a key role in kidney fibrosis development. Nat Med. 2015;21(1):37–46. doi: 10.1038/nm.3762 25419705 PMC4444078

[10] Kume S, Uzu T, Araki SI, et al. Role of altered renal lipid metabolism in the development of renal injury induced by a high-fat diet. J Am Soc Nephrol. 2007;18(10):2715–2723. doi: 10.1681/ASN.2007010089 17855643

[11] Declèves AE, Mathew AV, Armando AM, et al. Amp-activated protein kinase activation ameliorates eicosanoid dysregulation in high-fat-induced kidney disease in mice. J Lipid Res. 2019;60(5):937–952. doi: 10.1194/jlr.M088690 30862696 PMC6495162

[12] Yamamoto T, Takabatake Y, Takahashi A, et al. High-fat diet-induced lysosomal dysfunction and impaired autophagic flux contribute to Lipotoxicity in the kidney. *J Am Soc Nephrol*. 2016:1–18. doi: 10.1681/ASN.2016070731 PMC5407727 27932476

[13] Declèves AE, Zolkipli Z, Satriano J, et al. Regulation of lipid accumulation by AMK-Activated kinase in high fat diet-induced kidney injury. Kidney Int. 2014;85(3):611–623. doi: 10.1038/ki.2013.462 24304883 PMC4244908

[14] Juszczak F, Vlassembrouck M, Botton O, et al. Delayed exercise training improves obesity-induced chronic kidney disease by activating ampk pathway in high-fat diet-fed mice. Int J Mol Sci. 2021;22(1):1–20. doi: 10.3390/ijms22010350 PMC7795787 33396267

[15] Declèves AE, Mathew AV, Cunard R, et al. AMPK mediates the initiation of kidney disease induced by a high-fat diet. J Am Soc Nephrol. 2011;22(10):1846–1855. doi: 10.1681/ASN.2011010026 21921143 PMC3187184

[16] Dugan LL, You YH, Ali SS, et al. AMPK dysregulation promotes diabetes-related reduction of superoxide and mitochondrial function. J Clin Invest. 2013;123(11):4888–4899. doi: 10.1172/JCI66218 24135141 PMC3809777

[17] Li L, Wang C, Yang H, et al. Metabolomics reveal mitochondrial and fatty acid metabolism disorders that contribute to the development of DKD in T2DM patients. Mol Biosyst. 2017;13(11):2392–2400. doi: 10.1039/c7mb00167c 28956034

[18] Juszczak F, Pierre L, Decarnoncle M, et al. Sex differences in obesity-induced renal lipid accumulation revealed by lipidomics: a role of adiponectin/ampk axis. Biol Sex Differ. 2023;14(1):1–18. doi: 10.1186/s13293-023-00543-6 37770988 PMC10537536

[19] Juszczak F, Caron N, Mathew AV, et al. Critical role for AMPK in metabolic disease-induced chronic kidney disease. Int J Mol Sci. 2020;21(21):1–23. doi: 10.3390/ijms21217994 PMC7663488 33121167

[20] Mount P, Davies M, Choy SW, et al. Obesity-related chronic kidney disease—the role of lipid metabolism. Metabolites. 2015;5(4):720–732. doi: 10.3390/metabo5040720 26690487 PMC4693192

[21] Tang C, Livingston MJ, Liu Z, et al. Autophagy in kidney homeostasis and disease. Nat Rev Nephrol. 2020;16(9):489–508. doi: 10.1038/s41581-020-0309-2 32704047 PMC7868042

[22] Berquez M, Krohn P, Luciani A, et al. Receptor-mediated endocytosis and differentiation in proximal tubule cell systems. J Am Soc Nephrol. 2021;32(5):1265–1267. doi: 10.1681/ASN.2021020253 33846239 PMC8259691

[23] Park HW, Park H, Semple IA, et al. Pharmacological correction of obesity-induced autophagy arrest using calcium channel blockers. Nat Commun. 2014;5(1):1–12. doi: 10.1038/ncomms5834 PMC4157315 25189398

[24] Feldstein AE, Werneburg NW, Canbay A, et al. Free fatty acids promote hepatic lipotoxicity by stimulating tnf-α expression via a lysosomal pathway. Hepatology. 2004;40(1):185–194. doi: 10.1002/hep.20283 15239102

[25] Oh SJ, Hwang Y, Hur KY, et al. Lysosomal Ca2+ as a mediator of palmitate-induced lipotoxicity. Cell Death Discov. 2023;9(1). doi: 10.1038/s41420-023-01379-0 PMC10030853 36944629

[26] Jaishy B, Zhang Q, Chung HS, et al. Lipid-induced NOX2 activation inhibits autophagic flux by impairing lysosomal enzyme activity. J Lipid Res. 2015;56(3):546–561. doi: 10.1194/jlr.M055152 25529920 PMC4340303

[27] Zhang CS, Jiang B, Li M, et al. The lysosomal v-ATPase-ragulator complex is a common activator for AMPK and mTORC1, acting as a switch between catabolism and anabolism. Cell Metab. 2014;20(3):526–540. doi: 10.1016/j.cmet.2014.06.014 25002183

[28] Paquette M, El-Houjeiri L, Zirden LC, et al. Ampk-dependent phosphorylation is required for transcriptional activation of TFEB and TFE3. Autophagy. 2021;17(12):1–19. doi: 10.1080/15548627.2021.1898748 33734022 PMC8726606

[29] Fernandez-Mosquera L, Yambire KF, Couto R, et al. Mitochondrial respiratory chain deficiency inhibits lysosomal hydrolysis. Autophagy. 2019;15(9):1572–1591. doi: 10.1080/15548627.2019.1586256 30917721 PMC6693470

[30] Papadopoulos C, Kravic B, Meyer H. Repair or Lysophagy: dealing with damaged Lysosomes. J Mol Biol. 2020;432(1):231–239. doi: 10.1016/j.jmb.2019.08.010 31449799

[31] Tapper H, Sundler R. Bafilomycin a 1 inhibits lysosomal, phagosomal, and plasma membrane H+-ATPase and induces lysosomal enzyme secretion in macrophages. J Cell Physiol. 1995;144(1):137–144. doi: 10.1002/jcp.1041630116 7896890

[32] Oshima R, Hasegawa T, Tamai K, et al. ESCRT-0 dysfunction compromises autophagic degradation of protein aggregates and facilitates ER stress-mediated neurodegeneration via apoptotic and necroptotic pathways. Sci Rep. 2016;6(February):1–15. doi: 10.1038/srep24997 27112194 PMC4845015

[33] Kim J, Kundu M, Viollet B, et al. AMPK and mTOR regulate autophagy through direct phosphorylation of Ulk1. Nat Cell Biol. 2011;13(2):132–141. doi: 10.1038/ncb2152 21258367 PMC3987946

[34] González A, Hall MN, Lin SC, et al. AMPK and TOR: the yin and Yang of cellular nutrient sensing and growth control. Cell Metab. 2020;31(3):472–492. doi: 10.1016/j.cmet.2020.01.015 32130880

[35] Jang M, Park R, Kim H, et al. AMPK contributes to autophagosome maturation and lysosomal fusion. Sci Rep. 2018;8(1):1–10. doi: 10.1038/s41598-018-30977-7 30140075 PMC6107659

[36] Ducommun S, Ford RJ, Bultot L, et al. Enhanced activation of cellular AMPK by dual-small molecule treatment: AICAR and A769662. Am J Physiol - Endocrinol Metab. 2014;306(6):688–696. doi: 10.1152/ajpendo.00672.2013 PMC3948978 24425763

[37] Lamark T, Johansen T. Mechanisms of selective autophagy. Annu Rev Cell Dev Biol. 2021;37(1):143–169. doi: 10.1146/annurev-cellbio-120219-035530 34152791

[38] Singh R, Kaushik S, Wang Y, et al. Autophagy regulates lipid metabolism. Nature. 2009;458(7242):1131–1135. doi: 10.1038/nature07976 19339967 PMC2676208

[39] Mei S, Ni HM, Manley S, et al. Differential roles of unsaturated and saturated fatty acids on autophagy and apoptosis in hepatocytes. J Pharmacol Exp Ther. 2011;339(2):487–498. doi: 10.1124/jpet.111.184341 21856859 PMC3199993

[40] Varshney R, Varshney R, Mishra R, et al. Kaempferol alleviates palmitic acid-induced lipid stores, endoplasmic reticulum stress and pancreatic β-cell dysfunction through AMPK/mTOR-mediated lipophagy. J Nutr Biochem. 2018;57:212–227. doi: 10.1016/j.jnutbio.2018.02.017 29758481

[41] Pérez-Martí A, Ramakrishnan S, Li J, et al. Reducing lipid bilayer stress by monounsaturated fatty acids protects renal proximal tubules in diabetes. Elife. 2022;11:1–31. doi: 10.7554/eLife.74391 PMC9154741 35550039

[42] Vendruscolo M. Lipid homeostasis and its links with protein misfolding diseases. Front Mol Neurosci. 2022;15(March):1–13. doi: 10.3389/fnmol.2022.829291 PMC8990168 35401104

[43] Lamark T, Johansen T. Aggrephagy: selective disposal of protein aggregates by macroautophagy. Int J Cell Biol. 2012;2012:1–21. doi: 10.1155/2012/736905 PMC3320095 22518139

[44] Hernández-Cáceres MP, Cereceda K, Hernández S, et al. Palmitic acid reduces the autophagic flux in hypothalamic neurons by impairing autophagosome-lysosome fusion and endolysosomal dynamics. Mol Cell Oncol. 2020;7(5). doi: 10.1080/23723556.2020.1789418 PMC7469685 32944643

[45] Miyagawa K, Oe S, Honma Y, et al. Lipid-induced endoplasmic reticulum stress impairs selective autophagy at the step of Autophagosome-lysosome fusion in hepatocytes. Am J Pathol. 2016;186(7):1861–1873. doi: 10.1016/j.ajpath.2016.03.003 27157992

[46] Yagi M, Toshima T, Amamoto R, et al. Mitochondrial translation deficiency impairs NAD + ‐mediated lysosomal acidification. Embo J. 2021;40(8):1–17. doi: 10.15252/embj.2020105268 PMC8047443 33528041

[47] Kim HN, Seo BR, Kim H, et al. Cilostazol restores autophagy flux in bafilomycin A1-treated, cultured cortical astrocytes through lysosomal reacidification: roles of PKA, zinc and metallothionein 3. Sci Rep. 2020;10(1):1–12. doi: 10.1038/s41598-020-66292-3 32514052 PMC7280249

[48] Chauhan S, Kumar S, Jain A, et al. TRIMs and galectins globally cooperate and TRIM16 and galectin-3 Co-direct autophagy in endomembrane damage homeostasis. Dev Cell. 2016;39(1):13–27. doi: 10.1016/j.devcel.2016.08.003 27693506 PMC5104201

[49] Jia J, Claude-Taupin A, Gu Y, et al. Galectin-3 coordinates a cellular system for lysosomal repair and removal. Dev Cell. 2020;52(1):69–87.e8. doi: 10.1016/j.devcel.2019.10.025 31813797 PMC6997950

[50] Settembre C, Di Malta C, Polito VA, et al. TFEB links autophagy to lysosomal biogenesis. Science. 2011;332(6036):1429–1433. doi: 10.1126/science.1204592 21617040 PMC3638014

[51] Nielsen R, Christensen EI, Birn H. Megalin and cubilin in proximal tubule protein reabsorption: from experimental models to human disease. Kidney Int. 2016;89(1):58–67. doi: 10.1016/j.kint.2015.11.007 26759048

[52] Kim J, Kim SH, Kang H, et al. TFEB–GDF15 axis protects against obesity and insulin resistance as a lysosomal stress response. Nat Metab. 2021;3(3):410–427. doi: 10.1038/s42255-021-00368-w 33758420

[53] Liu Y, Steinbusch LKM, Nabben M, et al. Palmitate-induced vacuolar-type H±ATPase inhibition feeds forward into insulin resistance and contractile dysfunction. Diabetes. 2017;66(6):1521–1534. doi: 10.2337/db16-0727 28302654

[54] Maria Bagh DR, Chandra G, Peng S, et al. A lysosomal targeting defect of V0a1 suppresses V-ATPase activity elevating lysosomal pH in Ppt1-/- mice: amelioration by NtBuHA. Faseb J. 2015. https://faseb.onlinelibrary.wiley.com/doi/abs/10.1096/fasebj.29.1_supplement.570.6

[55] Bagh MB, Peng S, Chandra G, et al. Misrouting of v-ATPase subunit V0a1 dysregulates lysosomal acidification in a neurodegenerative lysosomal storage disease model. Nat Commun. 2017;8(1):8. doi: 10.1038/ncomms14612 28266544 PMC5344305

[56] Repnik U, Hafner Česen M, Turk B. Lysosomal membrane permeabilization in cell death: concepts and challenges. Mitochondrion. 2014;19(Part A):49–57. doi: 10.1016/j.mito.2014.06.006 24984038

[57] Wilson PD, Firestone RA, Lenard J. The role of lysosomal enzymes in killing of mammalian cells by the lysosomotropic detergent N-dodecylimidazole. J Cell Biol. 1987;104(5):1223–1229. doi: 10.1083/jcb.104.5.1223 3571330 PMC2114483

[58] Maclean KH, Dorsey FC, Cleveland JL, et al. Targeting lysosomal degradation induces p53-dependent cell death and prevents cancer in mouse models of lymphomagenesis. J Clin Invest. 2008;118(4):1584–1584. doi: 10.1172/JCI33700C1 PMC2148253 18097482

[59] Trudeau KM, Colby AH, Zeng J, et al. Lysosome acidification by photoactivated nanoparticles restores autophagy under lipotoxicity. J Cell Biol. 2016;214(1):25–34. doi: 10.1083/jcb.201511042 27377248 PMC4932370

[60] Zeng J, Acin-Perez R, Assali EA, et al. Restoration of lysosomal acidification rescues autophagy and metabolic dysfunction in non-alcoholic fatty liver disease. Nat Commun. 2023;14(1). doi: 10.1038/s41467-023-38165-6 PMC10160018 37142604

[61] Davis OB, Shin HR, Lim CY, et al. NPC1-mTORC1 signaling couples cholesterol sensing to organelle homeostasis and is a targetable pathway in niemann-pick type C. Dev Cell. 2021;56(3):260–276.e7. doi: 10.1016/j.devcel.2020.11.016 33308480 PMC8919971

[62] Bartolomeo R, Cinque L, De Leonibus C, et al. MTORC1 hyperactivation arrests bone growth in lysosomal storage disorders by suppressing autophagy. J Clin Invest. 2017;127(10):3717–3729. doi: 10.1172/JCI94130 28872463 PMC5617676

[63] Berquez M, Chen Z, Festa BP, et al. Lysosomal cystine export regulates mTORC1 signaling to guide kidney epithelial cell fate specialization. Nat Commun. 2023;14(1). doi: 10.1038/s41467-023-39261-3 PMC10349091 37452023

[64] Liu Y, Lai YC, Hill EV, et al. Phosphatidylinositol 3-phosphate 5-kinase (PIKfyve) is an AMPK target participating in contraction-stimulated glucose uptake in skeletal muscle. Biochem J. 2013;455(2):195–206. doi: 10.1042/BJ20130644 23905686

[65] Jin N, Lang MJ, Weisman LS. Phosphatidylinositol 3,5-bisphosphate: regulation of cellular events in space and time. Biochem Soc Trans. 2016;44(1):177–184. doi: 10.1042/BST20150174 26862203 PMC4836390

[66] McGuire CM, Forgac M. Glucose starvation increases V-ATPase assembly and activity in mammalian cells through AMP kinase and phosphatidylinositide 3-kinase/akt signaling. J Biol Chem. 2018;293(23):9113–9123. doi: 10.1074/jbc.RA117.001327 29540478 PMC5995505

[67] Nakamura S, Shigeyama S, Minami S, et al. LC3 lipidation is essential for TFEB activation during the lysosomal damage response to kidney injury. Nat Cell Biol. 2020;22(10):1252–1263. doi: 10.1038/s41556-020-00583-9 32989250

[68] Samie MA, Xu H. Thematic review series: recent advances in the treatment of lysosomal storage diseases: lysosomal exocytosis and lipid storage disorders. J Lipid Res. 2014;55(6):995–1009. doi: 10.1194/jlr.R046896 24668941 PMC4031951

[69] Medina DL, Fraldi A, Bouche V, et al. Transcriptional activation of lysosomal exocytosis promotes cellular clearance. Dev Cell. 2011;21(3):421–430. doi: 10.1016/j.devcel.2011.07.016 21889421 PMC3173716

[70] Klein D, Büssow H, Fewou SN, et al. Exocytosis of storage material in a lysosomal disorder. Biochem Biophys Res Commun. 2005;327(3):663–667. doi: 10.1016/j.bbrc.2004.12.054 15649398

[71] Kalluri R, Neilson EG, Neilson EG, et al. Epithelial-mesenchymal transition and its implications for fibrosis find the latest version: epithelial-mesenchymal transition and its implications for fibrosis. J Clin Investigation. 2003;112(12):1776–1784. doi: 10.1172/JCI200320530 PMC297008 14679171

[72] Iwano M, Plieth D, Danoff TM, et al. Evidence that fibroblasts derive from epithelium during tissue fibrosis. J Clin Invest. 2002;110(3):341–350. doi: 10.1172/JCI0215518 12163453 PMC151091

[73] Rovin BH, Adler SG, Barratt J, et al. KDIGO 2021 clinical practice guideline for the management of glomerular diseases. Kidney Int. 2021;100(4):S1–S276. doi: 10.1016/j.kint.2021.05.021 34556256

[74] Chagnac A, Zingerman B, Rozen-Zvi B, et al. Consequences of glomerular hyperfiltration: the role of physical forces in the pathogenesis of chronic kidney disease in diabetes and obesity. Nephron. 2019;143(1):38–42. doi: 10.1159/000499486 30947190

[75] Rampanelli E, Ochodnicky P, Vissers JPC, et al. Excessive dietary lipid intake provokes an acquired form of lysosomal lipid storage disease in the kidney. J Pathol. 2018;246(4):470–484. doi: 10.1002/path.5150 30073645

[76] Tojo A, Kinugasa S. Mechanisms of glomerular albumin filtration and tubular reabsorption. Int J Nephrol. 2012;2012:1–9. doi: 10.1155/2012/481520 PMC3363986 22685655

[77] Kumar S, Liu J, Pang P, et al. Sox9 activation highlights a cellular pathway of renal repair in the acutely injured mammalian kidney. Cell Rep. 2015;12(8):1325–1338. doi: 10.1016/j.celrep.2015.07.034 26279573

[78] Nakagawa S, Nishihara K, Miyata H, et al. Molecular markers of tubulointerstitial fibrosis and tubular cell damage in patients with chronic kidney disease. PLoS One. 2015;10(8):1–14. doi: 10.1371/journal.pone.0136994 PMC4552842 26317775

[79] Matsushita K, Toyoda T, Yamada T, et al. Specific expression of survivin, SOX9, and CD44 in renal tubules in adaptive and maladaptive repair processes after acute kidney injury in rats. J Appl Toxicol. 2021;41(4):607–617. doi: 10.1002/jat.4069 32969066

[80] Wang Z, Divanyan A, Jourd’heuil FL, et al. Vimentin expression is required for the development of emt-related renal fibrosis following unilateral ureteral obstruction in mice. Am J Physiol - Ren Physiol. 2018;315(4):F769–F780. doi: 10.1152/ajprenal.00340.2017 PMC6335003 29631355

[81] Terryn S, Jouret F, Vandenabeele F, et al. A primary culture of mouse proximal tubular cells, established on collagen-coated membranes. Am J Physiol - Ren Physiol. 2007;293(2):F476–F485. doi: 10.1152/ajprenal.00363.2006 17475898

[82] Bultot L, Jensen TE, Lai YC, et al. Benzimidazole derivative small-molecule 991 enhances AMPK activity and glucose uptake induced by AICAR or contraction in skeletal muscle. Am J Physiol - Endocrinol Metab. 2016;311(4):E706–E719. doi: 10.1152/ajpendo.00237.2016 27577855 PMC5241553

[83] Jadot M, Andrianaivo F, Dubois F, et al. Effects of methylcyclodextrin on lysosomes. Eur J Biochem. 2001;268(5):1392–1399. doi: 10.1046/j.1432-1327.2001.02006.x 11231291

