# Supplementary material for: AMPK protects proximal tubular epithelial cells from lysosomal dysfunction and dedifferentiation induced by lipotoxicity
Source: Autophagy. 2024 Dec 15;21(4):860–80. doi: 10.1080/15548627.2024.2435238 (PMC11925112; doi:10.1080/15548627.2024.2435238)
Supplement: revised supplemental R3.docx [file KAUP_A_2435238_SM0624.docx]

**Supplemental figures and tables**

**Table S1.** List of mouse oligonucleotides primers used in RT-qPCR.

| **Gene name** | **Forward(F)/Reverse (R)** | **Sequence (5’ to 3’)** |
| --- | --- | --- |
| *Acta2* | F | taacccttcagcgttcagc |
|  | R | acatagctggagcagcgtct |
| *Actb* | F | CTAAGGCCAACCGTGAAAAG |
|  | R | ACCAGAGGCATACAGGGACA |
| *Aqp1* | F | CCGAGACTTAGGTGGCTCAG |
|  | R | ATGCGGTCTGTAAAGTCGCT |
| *Cdh16* | F | CCAGCTCCCTCTGAACTCAC |
|  | R | CCCATGGTCATCCCATAAAG |
| *Cd44* | F | GTACGGAGTCAAATACCAACC |
|  | R | CAGCCATCCTGGTGGTTGTC |
| *Cd63* | F | GAAGCAGGCCATTACCCATGA |
|  | R | TGACTTCACCTGGTCTCTAAACA |
| *Cubn* | F | TTCCTTCTGGAGTGGTTTGC |
|  | R | cctgtcaccatataccctccac |
| *Dsp* | F | GTGATTCTGCAAGAGGCTGC |
|  | R | GCCAGTCTTAGCTCCTCTTCC |
| *Epb41l5* | F | ACGCTGCAAATGAAAGCCAG |
|  | R | GCAAATCAACGCTTAGGGGC |
| *Fn1* | F | GCGACTCTGACTGGCCTTAC |
|  | R | CCGTGTAAGGGTCAAAGCAT |
| *Foxm1* | F | AAGGCAAAGACAGGAGAGCT |
|  | R | AGGGCTCCTCAACCTTAACC |
| *Lamp1* | F | CAGCACTCTTTGAGGTGAAAAAC |
|  | R | ACGATCTGAGAACCATTCGCA |
| *Lcn2* | F | ctacaaccagttcgccatgg |
|  | R | acactcaccacccattcagt |
| *Lrp2* | F | tcaccagtgcctctgtgaag |
|  | R | agataatggaggccgcact |
| *Slc34a1* | F | GGGAGAAGCTATCCAGCTCA |
|  | R | ACAGCAAACCAGCGGTACTT |
| *Slc5a2* | F | TTGGGCATCACCATGATTTA |
|  | R | GCTCCCAGGTATTTGTCGAA |
| *Sox9* | F | CAAGAACAAGCCACACGTCA |
|  | R | GTGGTCTTTCTTGTGCTGCA |
| *Uvrag* | F | CTTCTGGATACCTACTTCAC |
|  | R | GACTTTCCACTCTATCAACAGC |
| *Vim* | F | AATGCTTCTCTGGCACGTCT |
|  | R | AGTGAGGTCAGGCTTGGAAA |
| *Vps11* | F | CAAGCCTACAAACTACGGGTG |
|  | R | GAGTGCAGAGTGGATTGCCA |
| *Vps18* | F | GCTCCGCATTGACTTGGG |
|  | R | GCCTTCTGTCCATTGCGG |

**
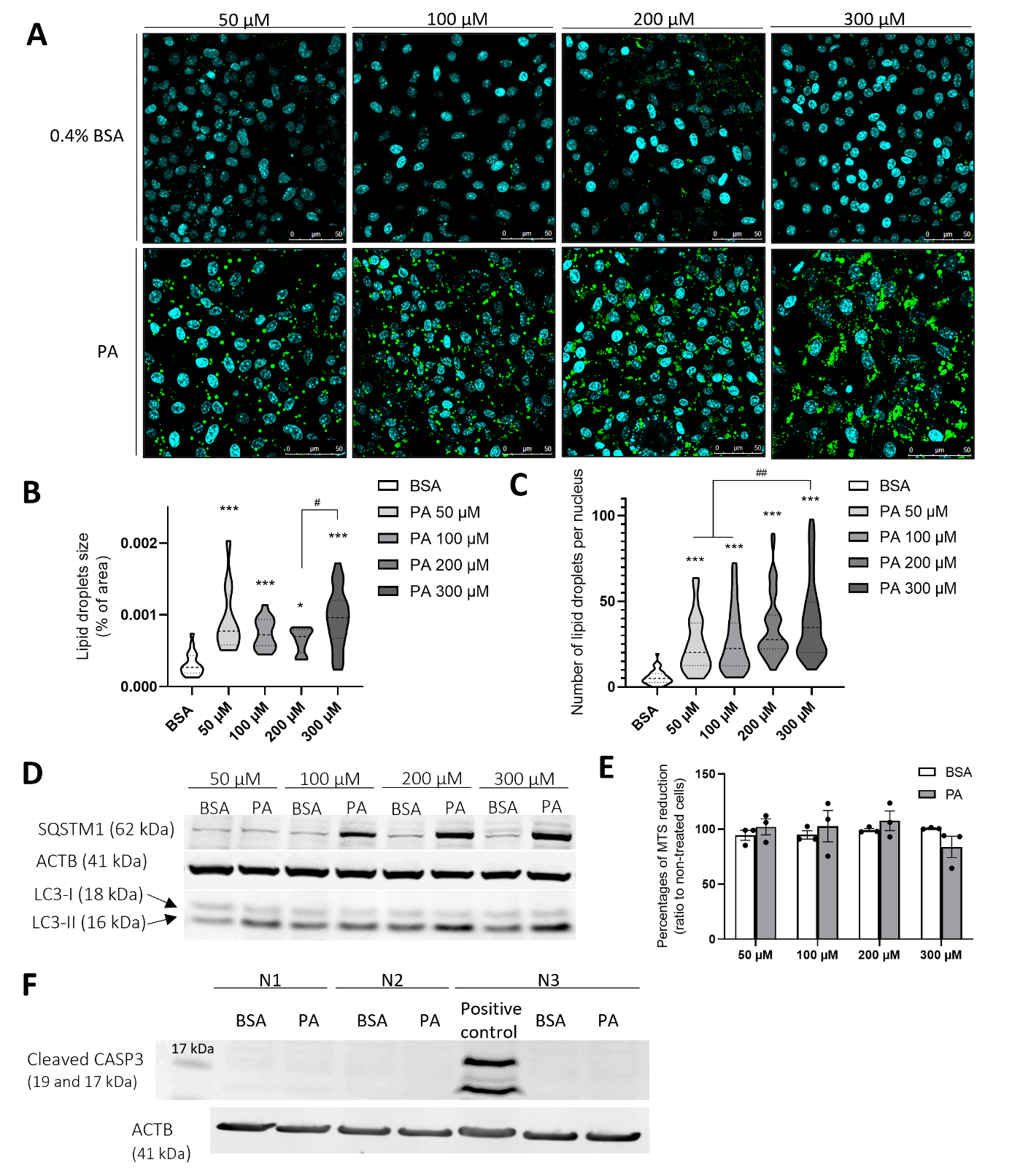
**

**Figure S1.** Modulation of autophagic markers and lipid droplets depends on PA concentration in MmPTECs. (**A**) MmPTECs were treated for 24 h with 50, 100, 200 or 300 µM PA or 0.4% BSA and stained with BODIPY^TM^ 493/503 for 15 min. (**B, C**) Quantifications of lipid droplet (**B**) number and (**C**) size on 100 cells per group by Lipid Droplets MRI tool. (**D**) Representative western blot of LC3, SQSTM1 and ACTB in MmPTECs treated with 50, 100, 200 or 300 µM PA or 0.4% BSA for 24 h. (**E**) Percentages of MTT reduction in MmPTECs treated with 50, 100, 200 or 300 µM PA or 0.4% BSA for 24 h. (**F**) Representative western blot of cleaved-CASP3 (caspase 3) and ACTB in MmPTECs treated with 300 µM PA or 0.4% BSA for 24 h or with 10 µM camptothecin for 4 h (positive control) of three independent biological replicates (N1, N2, N3). Data are presented as (**B, C**) means and quarters or as (**E**) means ± SEM of three independent biological experiments. Statistical analyses were performed by two-way ANOVA followed by Tukey post-hoc test. *p ≤ 0.05; **p ≤ 0.01; ***p ≤ 0.001 versus corresponding BSA group; #p ≤ 0.05; ## p ≤ 0.01; ### p ≤ 0.001 between PA groups.

**
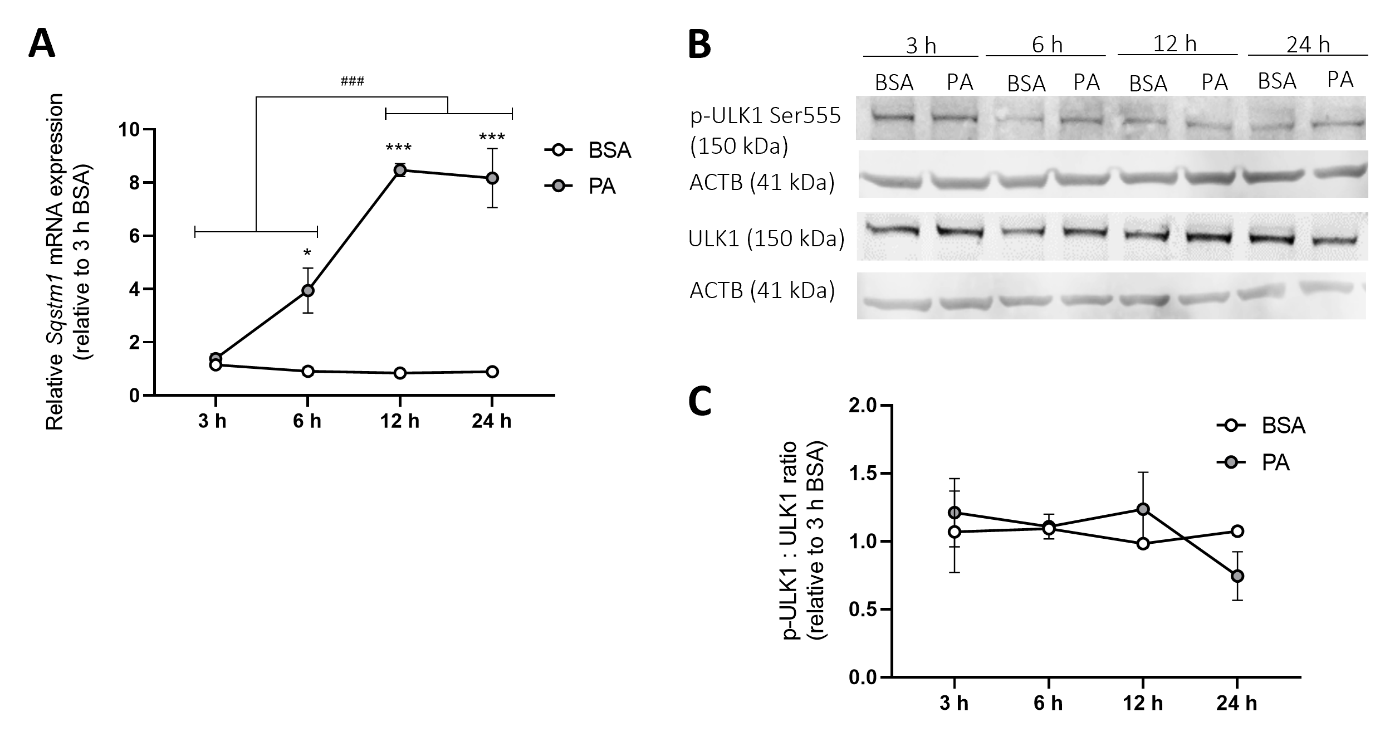
**

**Figure S2.** PA significantly upregulates *Sqstm1* expression but does not affect the phosphorylation of ULK1 in MmPTECs. (**A**) Relative mRNA expression of *Sqstm1* on MmPTEC treated with 300 µM PA or 0.4% BSA for 3, 6, 12 or 24 h. (**B**) Representative western blot of of p-ULK1 (Ser555), ULK1 and ACTB in MmPTECs treated with 300 µM PA or 0.4% BSA for 3, 6, 12 or 24 h. (**C)** Quantitative densitometry analysis of the p-ULK1:ULK1 ratio. Data are presented as means ± SEM of three independent biological experiments. Statistical analyses were performed by two-way ANOVA followed by Tukey post-hoc test. *p ≤ 0.05; ***p ≤ 0.001 vs corresponding BSA group; ###p ≤ 0.001 between PA groups.


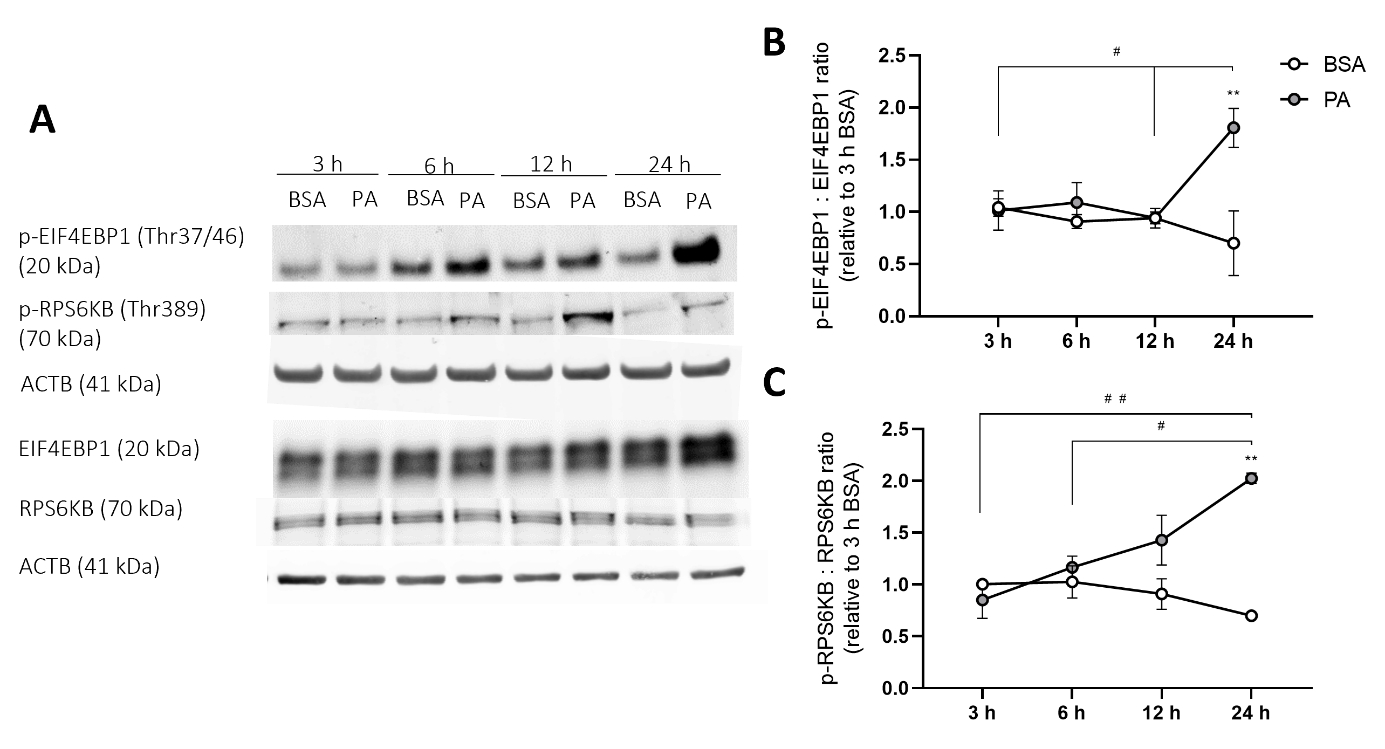


**Figure S3.** PA enhances MTORC1 activity and signalization after 24 h in MmPTECs. (**A**) Representative western blot of p-EIF4EBP1 (Thr37/46), p-RPS6KB (Thr389), EIF4EBP1, RPS6KB and ACTB in MmPTECs treated with 300 µM PA or 0.4% BSA for 3, 6, 12 or 24 h. (**B**, **C**) Quantitative densitometry analysis of the (**B**) p-EIF4EBP1:EIF4EBP1 ratio and (**C**) the p-RPS6KB:RPS6KB ratio. Data are presented as means ± SEM of three independent biological experiments. Statistical analyses were performed by two-way ANOVA and Tukey post-hoc test. **p ≤ 0.01 *versus* corresponding BSA group; #p ≤ 0.05; ##p ≤ 0.01 between PA groups.

**
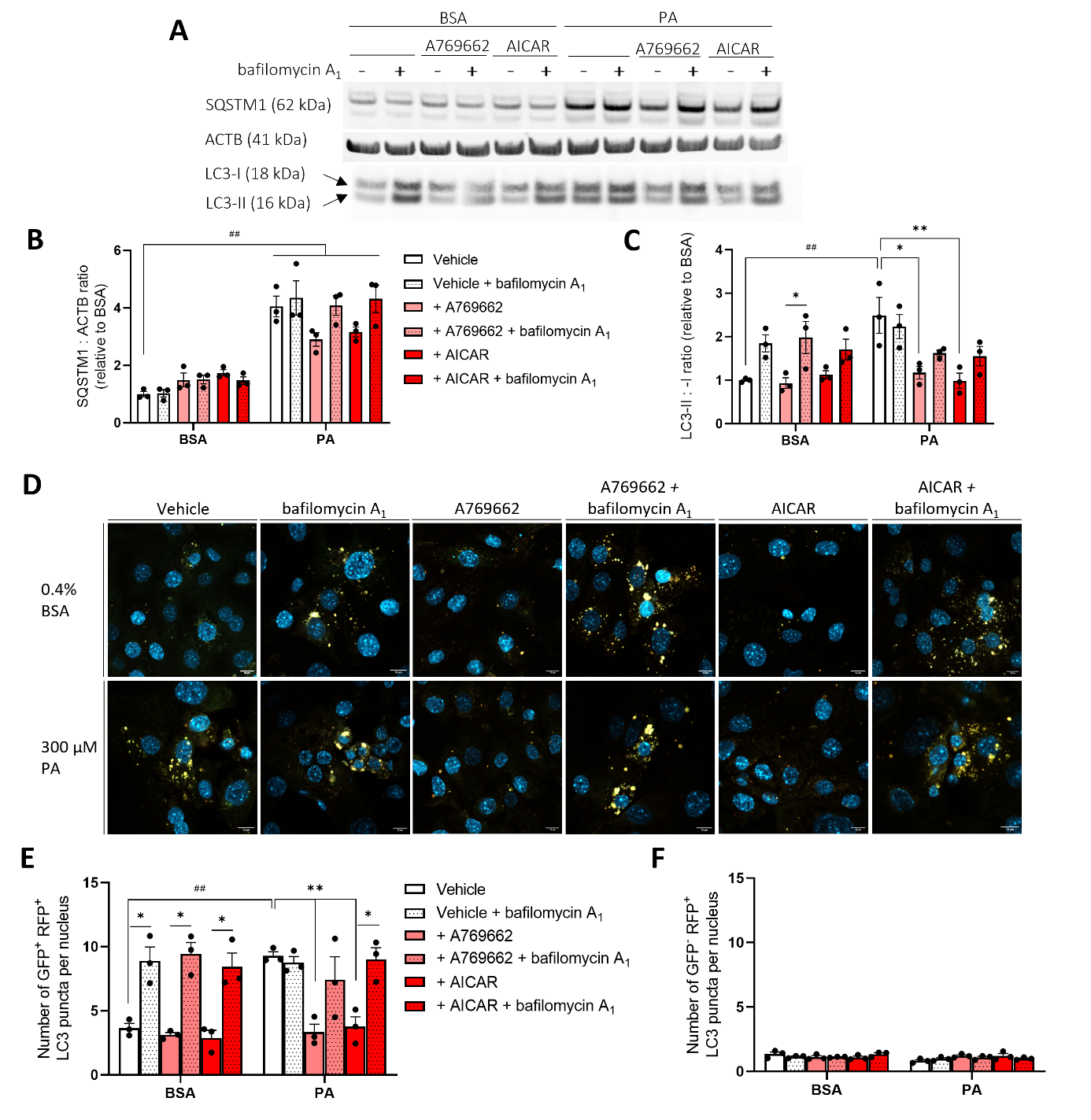
**

**Figure S4.** Six h of AMPK activation prevents PA-induced autophagosomes accumulation in MmPTECs. (**A**) Representative western blot and (**B, C**) quantitative densitometry analysis of LC3, SQSTM1 and ACTB in MmPTECs treated for 6 h with 300 µM PA or 0.4% BSA, in the presence of 100 µM A769662 or 2 mM AICAR and with or without 2 nM bafilomycin A_1_. (**D**) Representative micrographs of MmPTECs expressing mRFP-GFP-LC3B and treated for 6 h in the same conditions. GFP^+^ RFP^+^ (yellow) puncta indicate autophagosomes (neutral pH), and GFP^-^ RFP^+^ (red) ones indicate acidic pH. Quantifications of the number of (**E**) GFP^+^ RFP^+^ and (**F**) GFP^-^ RFP^+^ puncta on 30 cells per group. Data are presented as means ± SEM of three independent biological experiments. Statistical analyses were performed by two-way ANOVA and Tukey’s post-hoc test. *p ≤ 0.05; **p ≤ 0.01; ***p ≤ 0.001 in PA or BSA treatment groups; #p ≤ 0.05; ###p ≤ 0.001 between groups.


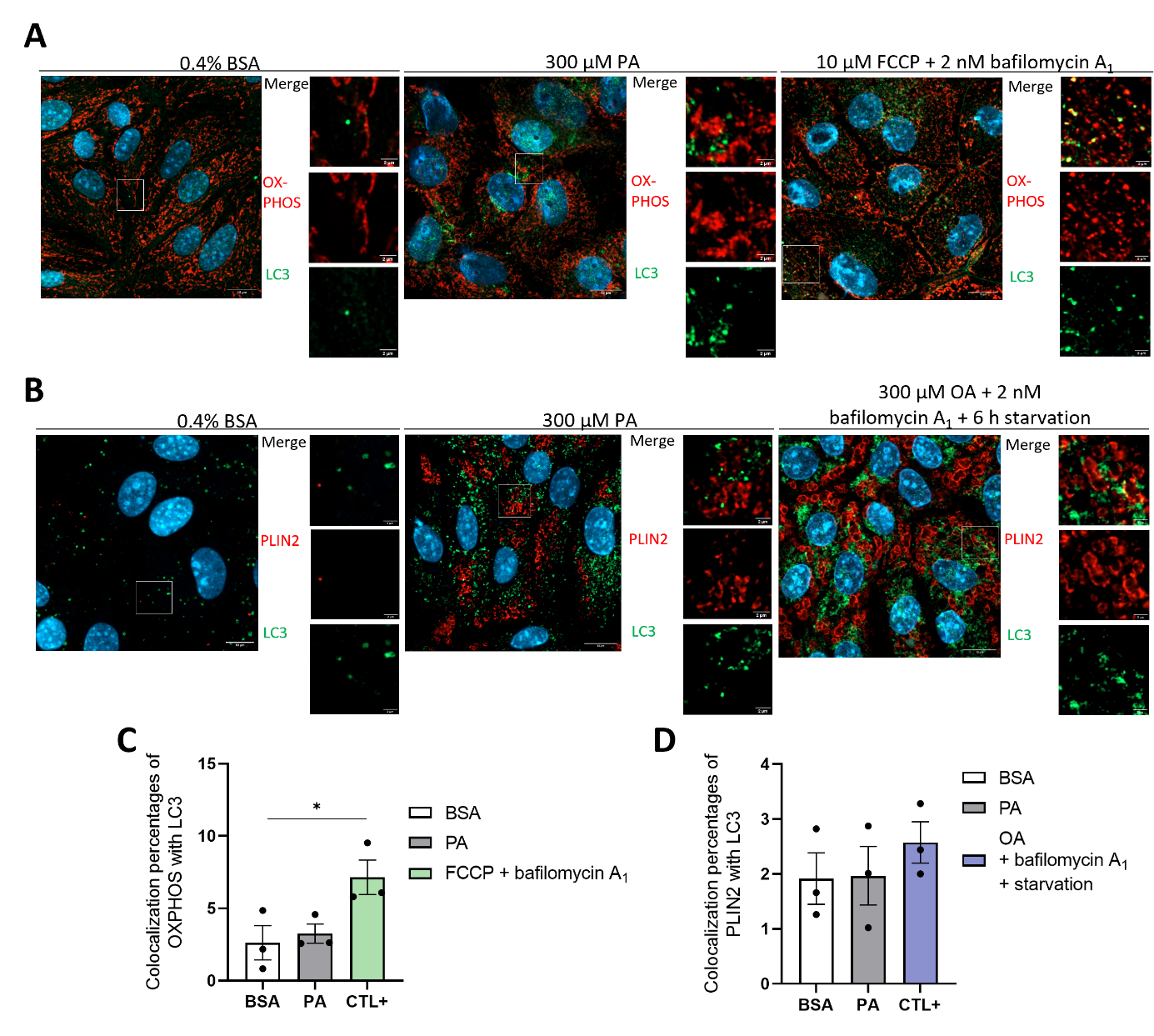


**Figure S5.** Autophagosomes in response to PA are negative for lipid droplet and mitochondrial markers in MmPTECs. (**A**) Representative micrographs of cells treated for 24 h with 300 µM PA, 0.4% BSA or for 6 h with 10 µM FCCP and 2 nM bafilomycin A_1_ (positive control), fixed and immuno-stained for OXPHOS (red) and LC3 (green). (**B**) Representative micrographs of MmPTECs treated for 24 h with 300 µM PA, 0.4% BSA or 300 µM OA and then starved in presence of 2 nM bafilomycin A_1_ for 6 h (positive control), fixed and immuno-stained for PLIN2 (red) and LC3 (green). (**C**, **D**) Quantifications of the colocalization percentages of (**C**) OXPHOS with LC3 and (**D**) PLIN2 with LC3 calculated by Manders’s correlation coefficients on 30 cells per group. Data are presented as means ± SEM of three independent biological experiments. Statistical analyses were performed by one-way ANOVA followed by Dunnett’s post-hoc test. *p ≤ 0.05 *versus* corresponding BSA groups.


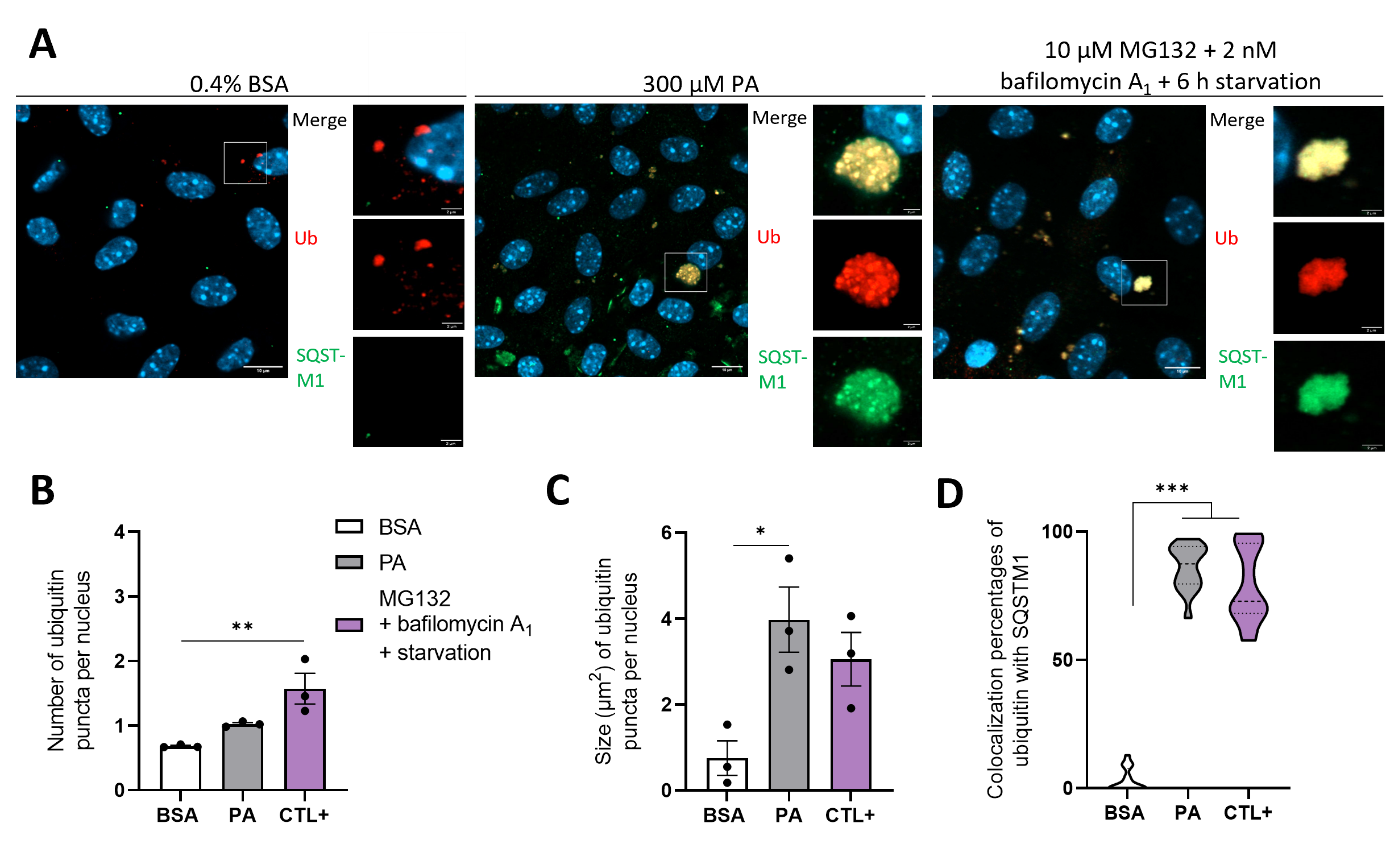


**Figure S6.** PA-induced accumulating autophagosomes contain ubiquitin-positive aggregates after 6 h in MmPTECs. (**A**) Representative micrographs of cells treated for 6 h with 300 µM PA, 0.4% BSA or with 10 µM MG132 and 2 nM bafilomycin A_1_ (positive control), fixed and immuno-stained for ubiquitin (red) and SQSTM1 (green). (**B**) Quantifications of the number of ubiquitin puncta per nucleus on 30 cells per group. (**C**) Quantification of the colocalization percentages of ubiquitin with SQSTM1 calculated by Manders’s correlation coefficients on 30 cells per group. Data are presented as (**B**, **C**) means ± SEM or as (**D**) means and quarters of three independent biological experiments as indicated on the charts. Statistical analyses were performed by one-way ANOVA followed by Dunnett’s post-hoc test. *p ≤ 0.05; **p ≤ 0.01; ***p ≤ 0.001 *versus* corresponding BSA groups.

**
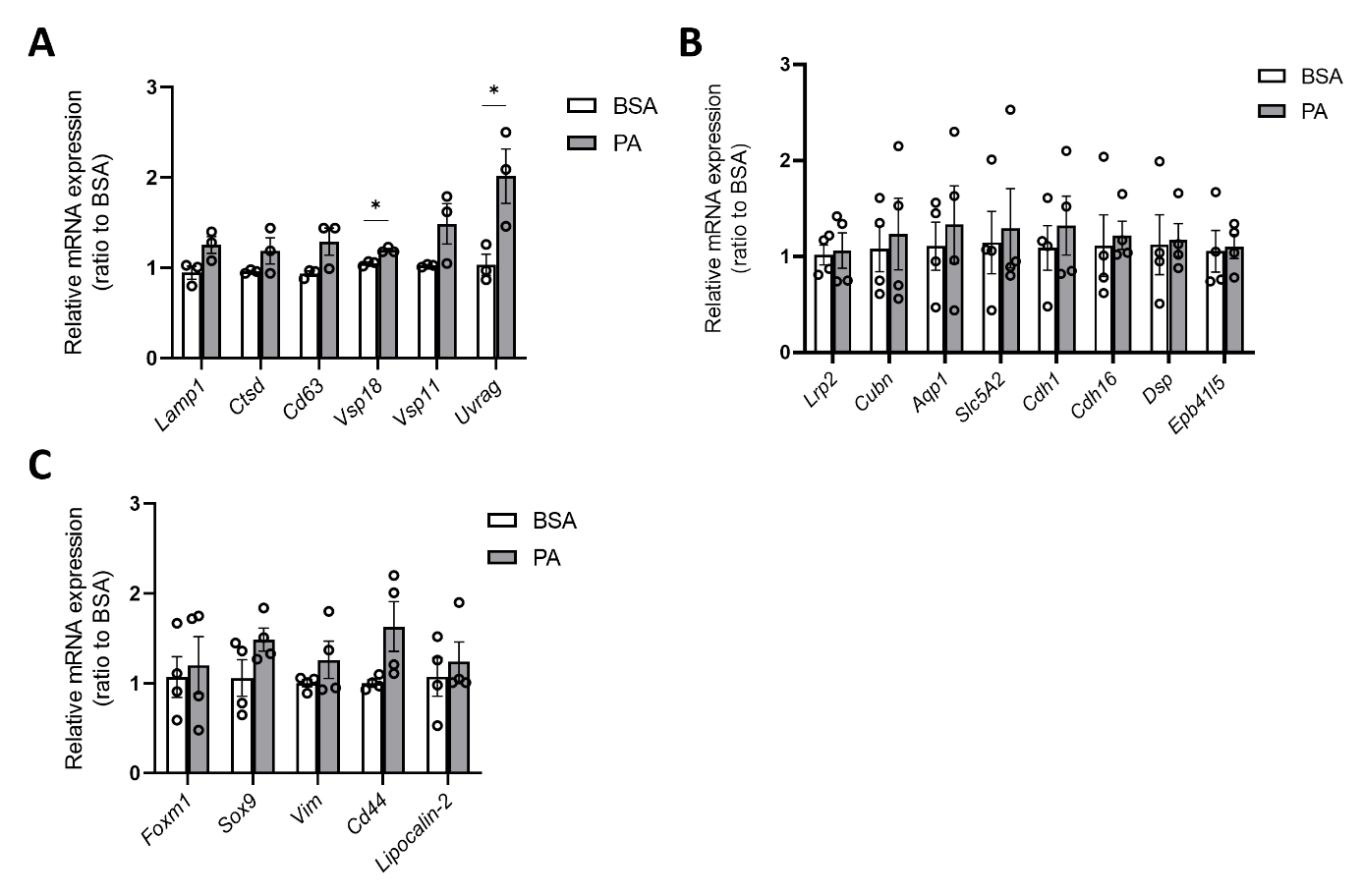
**

**Figure S7.** 6 h of PA treatment does not modify expression of lysosomal markers, differentiation nor dedifferentiation markers in MmPTECs. (**A**) Relative mRNA expression of TFEB-targeted genes on MmPTECs treated with 300 µM PA or 0.4% BSA for 6 h. (**B**, **C**) Relative mRNA expression of (**B**) differentiation and (**C**) dedifferentiation markers on MmPTECs treated with 300 µM PA or 0.4% BSA for 6 h. Data are presented as means ± SEM of three or four independent biological experiments as indicated on the charts. Statistical analyses were performed by Student’s unpaired t-test. *p ≤ 0.05 *versus* corresponding BSA groups.

**
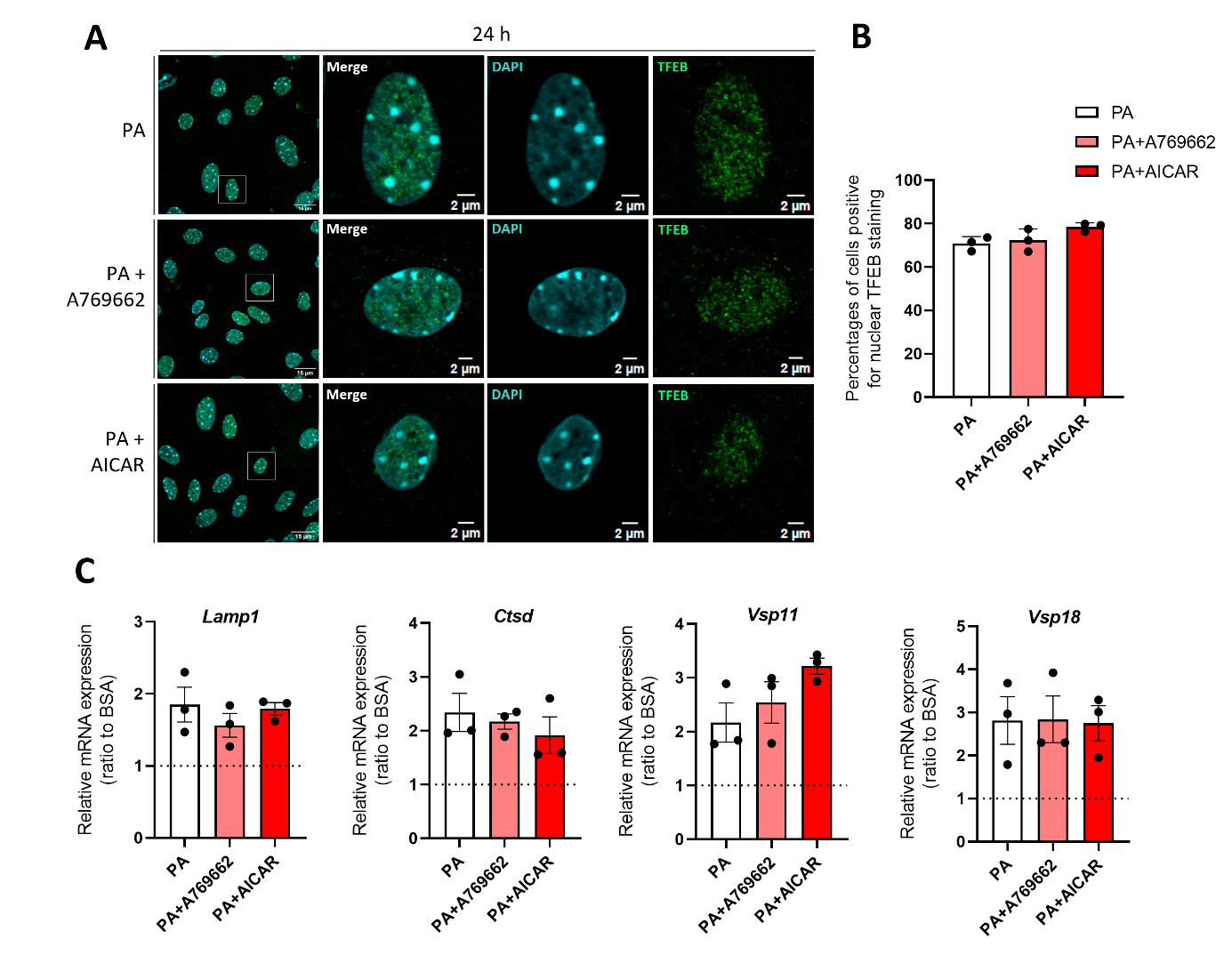
Figure S8.** Pharmacological AMPK activation does not prevent lysosomal biogenesis in PA-treated MmPTECs. (**A**) Representative micrographs of cells treated for 24 h with 300 µM PA in the presence or absence of 100 µM A769662 or 2 mM AICAR, fixed and immuno-stained for TFEB (green). (**B**) Quantification of the percentages of cells showing nuclear TFEB staining on more than 100 cells per group. (**C**) Relative mRNA expression of TFEB-targeted genes on cells treated for 24 h with 300 µM PA in the presence or absence of 100 µM A769662 or 2 mM AICAR. Data are presented as means ± SEM of three independent biological experiments. Statistical analyses were performed by one-way ANOVA followed by Dunnett’s post-hoc test.
